# Possibilities for IPM Strategies in European Laying Hen Farms for Improved Control of the Poultry Red Mite (*Dermanyssus gallinae*): Details and State of Affairs

**DOI:** 10.3389/fvets.2020.565866

**Published:** 2020-11-17

**Authors:** Eva Decru, Monique Mul, Alasdair J. Nisbet, Alejandro H. Vargas Navarro, Geoffrey Chiron, Jon Walton, Tomas Norton, Lise Roy, Nathalie Sleeckx

**Affiliations:** ^1^Experimental Poultry Centre, Geel, Belgium; ^2^Wageningen Livestock Research, Division Animal Health and Welfare, Wageningen, Netherlands; ^3^MoniqueMul IPM, Wervershoof, Netherlands; ^4^Vaccines and Diagnostics Department Moredun Research Institute, Midlothian, United Kingdom; ^5^Koppert BV, Berkel en Rodenrijs, Netherlands; ^6^Technical Poultry Institute, Paris, France; ^7^RSK ADAS, Ltd., Bristol, United Kingdom; ^8^Group of M3-BIORES, Division of Animal and Human Health Engineering (A2H), Department of BioSystems, Katholieke Universiteit Leuven, Leuven, Belgium; ^9^CEFE, CNRS, University of Montpellier, University of Paul Valéry Montpellier, EPHE, IRD, Montpellier, France

**Keywords:** *Dermanyssus gallinae*, integrated pest management, poultry red mite, sustainable control, prevention, non-chemical, monitoring, layer houses

## Abstract

The Poultry Red Mite (PRM), *Dermanyssus gallinae*, is a major threat to the poultry industry worldwide, causing serious problems to animal health and welfare, and huge economic losses. Controlling PRM infestations is very challenging. Conventionally, *D. gallinae* is treated with synthetic acaricides, but the particular lifestyle of the mite (most of the time spent off the host) makes the efficacy of acaracide sprays often unsatisfactory, as sprays reach only a small part of the population. Moreover, many acaricides have been unlicensed due to human consumer and safety regulations and mites have become resistant to them. A promising course of action is Integrated Pest Management (IPM), which is sustainable for animals, humans and the environment. It combines eight different steps, in which prevention of introduction and monitoring of the pest are key. Further, it focusses on non-chemical treatments, with chemicals only being used as a last resort. Whereas IPM is already widely applied in horticulture, its application is still in its infancy to control *D. gallinae* in layer houses. This review presents the currently-available possibilities for control of *D. gallinae* in layer houses for each of the eight IPM steps, including monitoring techniques, established and emerging non-chemical treatments, and the strategic use of chemicals. As such, it provides a needed baseline for future development of specific IPM strategies, which will allow efficient and sustainable control of *D. gallinae* in poultry farms.

## Introduction

**Figure 1:**
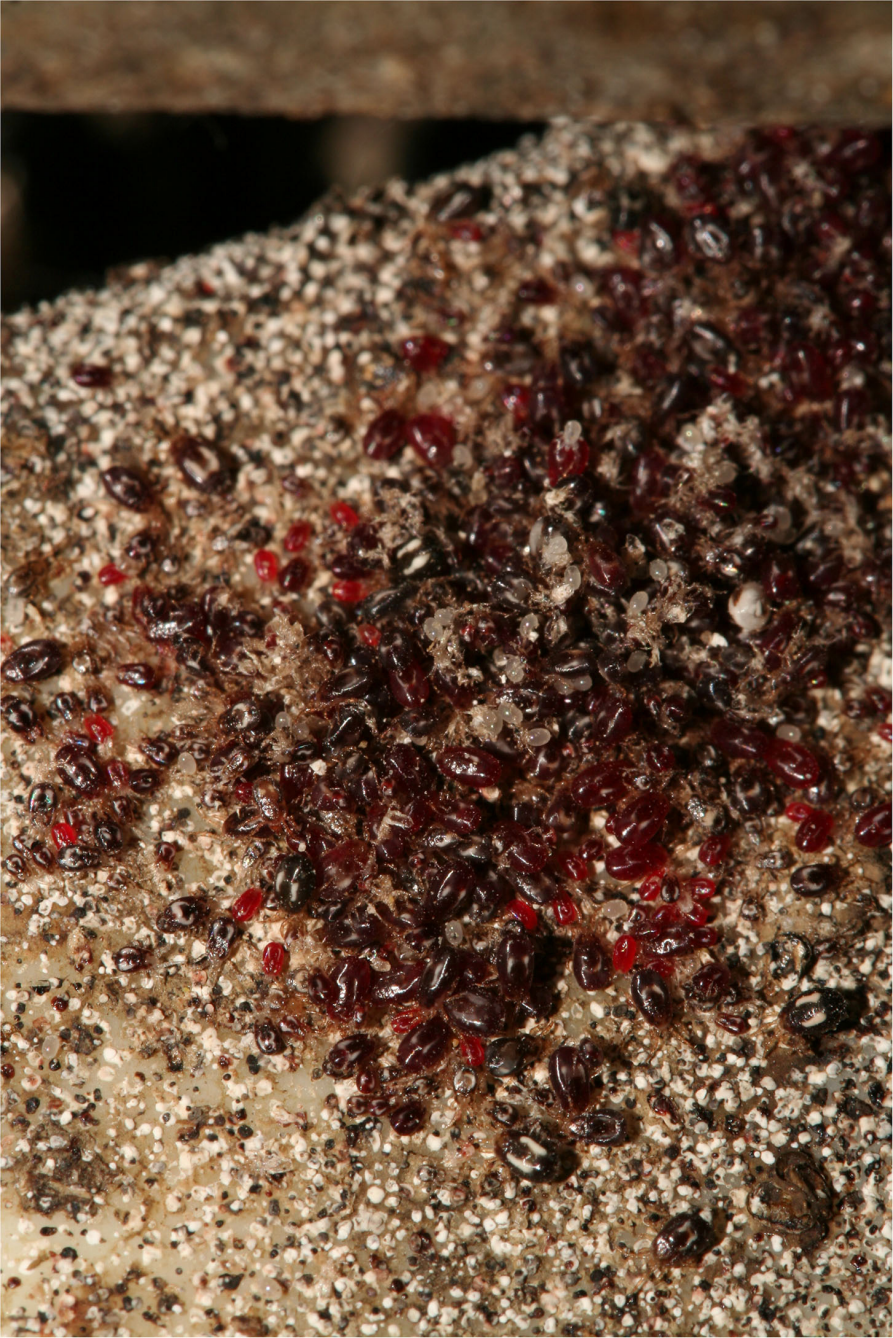
Photograph of an *in situ* mite (*Dermanyssus galllinae*) aggregate, composed of different stages of *D. gallinae* more or less freshly fed. The mite droppings (black and white marks surrounding the aggregate) form fairly persistent marks on structural elements in the farms, lasting long after an infestation. Photo credit: Rumsais Blatrix (CEFE/CNRS).

The Poultry Red Mite (PRM), *Dermanyssus gallinae* (De Geer, 1778) (Figure 1), is the most common ectoparasite belonging to the order of Mesostigmata and class of Arachnida (1) in poultry farming. A taxonomic key and high-resolution micrographs are available for a correct identification of the species (2, 3). It is a blood-sucking ectoparasite of laying hens, living off the host and mainly hiding in cracks and crevices near the hen's nightly resting place, therefore out of reach of the hens (4, 5). However, as it is a strictly hematophagous mite, it needs to find a hen from time to time to obtain a fast blood meal, which it does during the dark hours (6).

*Dermanyssus gallinae* is the most damaging ectoparasite of laying hens worldwide, particularly in Europe where the infestation rate of premises is well over 80% and in some countries even reaching 94% (7). Due to relatively high temperature and humidity, environmental conditions in a layer house are in general very favorable for *D. gallinae*. In such favorable conditions, the reproduction cycle of *D. gallinae*, can be completed in only 7 days, which often leads to a rapid exponential accumulation to very high numbers in a short period of time (8, 9). Infestations of *D. gallinae* pose serious threats to the welfare of the hens. They may cause restlessness, lack of sleep, stress, severe feather pecking, aggression, anemia and sometimes even death (1, 5, 10–13). Further, *D. gallinae* also poses a threat to human health, as it can act a reservoir and possibly as a vector of several zoonotic diseases like *Salmonella enteritidis, Pasteurella multocida*, and *Borrelia burgdorferi* (8, 11). The ectoparasite also increasingly causes human dermatological lesions in poultry handlers and urban citizens, which is currently still largely underdiagnosed (7, 12, 14). Infestations of *D. gallinae* also lead to huge economic losses. First of all, there is an increased mortality. Further, infested flocks have a higher feed and water conversion due to poor feathering and loss of blood. Despite the increased feed intake, hens will be lighter and less energy will go to the eggs, leading to lower production and lower egg weight. In addition, the percentage of second quality eggs increases due to a.o. blood spots (15, 16). Based on literature and field experience, Van Emous (2005) estimated the economic damage of *D. gallinae* infestations. He adapted these figures in 2017 (Table 1), as the situation changed mainly due to alternative housing systems, extended production cycles, and ban on beak-trimming in several countries (leading to more severe pecking impact). Including treatment costs, he estimated an economic damage of 31 million euros per year associated with *D. gallinae* in Europe.

**Table 1:** Estimation of economic impact at different levels of infestations of *D. gallinae*: medium (mites not visible); severe (mites visible); and very severe (many clusters visible on the system), according to (17), and updated for a severe infestation by Van Emous (15).

	**None**	**Medium**	**Severe**	**Very severe**
Feed intake (g/day/hen)	108	+0	+2	+2
Egg weight (g)	62	−0.2	−1	−1
Hen weight (g)	1,800	−25	−100	−100
Second q. egg (%)	6	+2	+6	+14
Mortality (%)	7	+0	+2	+5
Number of eggs (per hen housed)	345	−0	−3	−10

## Current Control Strategies

Conventional treatment of *D. gallinae* is dominated by the use of synthetic acaricides. However, there is currently a very limited amount of chemical acaricides available for use, as many have been withdrawn from the European market due to human consumer and user safety regulations (8). Further, increasing levels of resistance to commonly applied acaricides has been found in *D. gallinae*, causing lower efficacy of these products (18). The solitary use of chemicals is thus an end-of pipeline solution which is not regarded as sustainable (19). Much research has been done, and is still being done on the use of alternative non-chemical treatments, like plant-based products, natural predators and vaccines (see below *Non-chemical Treatment Methods*). However, none of these seem to be efficient enough to serve as a stand-alone treatment against *D. gallinae* (16). The only sustainable solution to control *D. gallinae* infestation is to use multi-tactic Integrated Pest Management programmes, as already suggested by several authors (5, 20–23). However, in practice, use of this approach in the poultry industry is still very limited and merely restricted to a combination of biosecurity measures, chemical acaricides and physical treatments in-between flocks (9, 23).

## Integrated Pest Management

Integrated Pest Management (IPM) is a strategy to control pest species, which is sustainable for animals, humans, and the environment. IPM consists of eight steps, in which prevention of introduction, and monitoring of the pest are key for sustainable control (24). For successful IPM, all ecological and biological knowledge, including biotic and abiotic factors, of the pest species should be integrated. Monitoring is crucial to identify the best moment for applying treatments. Principally, environmentally-safe, non-chemical methods and measures are used for prevention and control of the pest species. Only when non-chemical measures have failed and an action threshold is exceeded is a chemical treatment deployed as a last resort. Preferably, a selective acaricide should be used in order to avoid killing non-target species, and the use of chemicals should be in an as limited way as possible (e.g., hot-spot treatments). Actions to avoid resistance against products should be implemented, and finally, thorough evaluation of the IPM strategy is needed to optimize it.

In horticulture, IPM is currently well-advanced and widely applied. Already since 1959, the International Organization for Biological and Integrated Control (IOBC) has formed international, multidisciplinary teams, to examine methods for biological controls, each team focusing on a particular pest. Through the work of the IOBC, specific IPM strategies for all major crops have been developed. Currently, all EU countries need to have an IPM action plan for these crops (25).

At present, IPM is primarily used to control plant pests, and the practical implementation of IPM in animal husbandry is in its infancy compared to horticulture. Monitoring is only applied in a minority of farms and, in the use of non-chemical alternatives, livestock farming is lagging far behind. Indeed, the definition of biocontrol on governmental or academic webpages often only address plant protection [e.g., (26)].

The potential of IPM in livestock production has already been discussed in 1981 by Axtell. Control of flies, ticks and worms via IPM in dairy farming and pig production is suggested and discussed in several studies (27, 28). Axtell (29) also suggested IPM to control poultry pests, and specifically for the control of *D. gallinae*, IPM has been suggested in several works (5, 8, 19). However, practical implementation is limited to only combining certain treatments (30).

A lot of synergies exist between the arthropod pest control in horticulture and the control of *D. gallinae* in layer farms, so we believe that the principles of IPM can also be applied in the poultry industry. In horticulture, multiple pests are generally tackled simultaneously with IPM strategies. In European layer houses, however, *D. gallinae* seems to be the only problematic ectoparasite, and other targeted pests are mainly flies and lesser mealworm (5). In the US, another problematic ectoparasite in layer houses is the Northern Fowl Mite *Ornithonyssus sylviarum* (Canestrini and Fanzago, 1877), but this pest does not seem to occur in European layer houses (31). Therefore, in contrast to the multiple species targeted in horticulture, the current review focusses on the control of *D. gallinae* solely. In the following sections, we provide an overview and discussion of the currently-available possibilities for each of the eight IPM steps for controlling *D. gallinae* in layer houses, and the still existing deficiencies that limit the delineation and implementation of IPM plans. As such, it provides a needed baseline for researchers to develop valuable research programs for advancing implementation of IPM strategies for control of *D. gallinae*.

## Prevention And Population Suppression (Step 1)

Infestations of *D. gallinae* do not only occur in layer farms but also in the breeder flocks and rearing farms, and *D. gallinae* can survive and spread through pullet, egg and manure transport (19, 32).

In layer houses, the very first step in the IPM approach is thus preventing new populations of *D. gallinae* from entering and spreading in the layer houses. Good biosecurity measures are considered highly beneficial for pest control, including *D. gallinae* (33–35).

In 2009, the Hazard Analysis and Critical Control Points (HACCP) method was used as a method for assessing risks in introduction and spread of *D. gallinae* in poultry farms (36). A total of 41 possible hazards for *D. gallinae* infection and spread have been identified by experts, 31 of them being identified as Critical Control Points (CPPs), i.e., points, steps or procedures with a high risk of mite infection and/or spread [(36), Table 2 therein]. These CCPs should be monitored carefully, and possible corrective actions should be undertaken when necessary. The influence of wild birds as a reservoir, as mentioned in this checklist, is, however, currently obsolete, and transmission of *D. gallinae* most probably occurs through the exchange of contaminated material or birds between farms or facilities (37, 38).

Although little information is to be found in the scientific literature, some practical guidelines [brochures and checklists, e.g., (39, 40), MSD Animal Health] for good hygiene and other biosecurity measures are available to poultry farmers for preventing infestation of *D. gallinae* and suppressing its population growth. In a recent project (41) on the control of *D. gallinae*, much attention is payed to these preventive and suppressive measures. The most important measures [based on (41)], are further discussed in the following paragraphs.

### Preventing *D. Gallinae* From Entering and Spreading in Laying Hen Facilities

It has been demonstrated that commercial exchanges of contaminated birds and material are strongly involved in the spread of *D. gallinae* in layer farms (37, 38). There are several ways through which *D. gallinae* can enter and disperse throughout the facilities (also between layer houses if there are multiple at the farm). Good biosecurity measures can, however, considerably reduce these chances. Further, existing populations should be prevented to grow to substantial numbers. Preventive measures need to be taken into account during the production as well as between laying rounds, but also when building/installing new facilities.

#### Between Laying Rounds

When layer houses are empty, hiding places for *D. gallinae*, which cannot be reached easily in the presence of hens, can be cleaned thoroughly. Cleaning with hot water and soap is strongly advised over only dry-cleaning the facilities. Table 2 illustrates all the steps that should ideally be undertaken, preferably in that order.

**Table 2:** Cleaning actions to be executed during the empty period for optimal control of *D. gallinae* infestations in a layer house according to Mul et al. (41).

	**Actions (preferable executed in this order)**
1	Remove manure
2	Remove all clustered manure residues (scraping)
3	Dry clean house (e.g., broom and remove all detritus and dirt)
4	Clean with compressor (also in pvc tubes and cable ties)
5	Clean air mixing box
6	Dry clean hen house second time
7	Clean ventilation duct (preferably with steam cleaner)
8	Clean aeration tubes (possibly by sewer cleaning company)
9	Clean manure belts
10	Clean central manure belts
11	Clean egg belts with high water pressure
12	Remove all dirt from the house
13	Clean whole house with steam cleaner
14	Let everything dry
15	Clean manure container/pit
16	Disinfect after drying

Other preventive treatments between flocks can be heat treatment alone or used in combination with inert substances (see further). Heat treatment cannot be used during production as it affects the hens, and thus can only be applied in-between flocks. A temperature of over 45°C is lethal for *D. gallinae* (19, 42). With the Thermokill method, layer houses are gradually heated up to over 45°C for at least 2 days (43). By heating up the layer house gradually, mites are also lured out of their hiding places. This way they are also more reachable with contact treatments (44). Preliminary findings from a field trial showed a good efficacy of applying heat treatment followed by a silica treatment, with strongly reduced infestation in the next flock (41). A downside of heat treatment seems to be that not all housing systems can resist the high temperatures, resulting in damaged structures. In addition, heat treatment is rather expensive, certainly for larger infrastructures with outdoor facilities for the hens, where heating evenly is difficult (19).

Acaricidal chemicals can also be sprayed during the empty period, but in the framework of IPM, chemical/synthetic treatments should not be used preventively; they should only be used when non-chemical preventive and curative treatments do not act sufficiently.

A project where in 20 commercial layer farms *D. gallinae* was monitored for 10 months (45) showed that the point at which *D. gallinae* is noticed for the first time during a flock is highly influenced by the cleaning and treatments during the empty period. In farms where a combination of dry cleaning, wet cleaning and some form of treatment (silica, plant-based oils, heat treatment or chemical treatments) was applied, a later re-infestation of *D. gallinae* was found compared to the other farms with less stringent regimes.

#### During Production

Mites are primarily dispersed into the facilities through the actions of people. In a poultry farm, several different people often need to enter the facilities (care-takers, farm managers, veterinarians, etc.). A first measure is to keep the number of external visitors limited and to apply strict hygiene regulations (e.g., wearing company clothing and hair nets, using hygiene barriers, wear separate boots in separate houses) for staff members and visitors. It is also important that delivered pullets and the crates in which they are delivered are mite-free. It is strongly advised that pullet breeders also monitor infestations in their facilities and share their monitoring data with the egg producer. It is also important that egg-trays and egg-containers, which are regularly brought in and out the facilities, are free of mites. Using disposable cardboard egg-trays is preferred. *Dermanyssus gallinae* can also disperse via vertebrates other than hens. A good pest-control strategy (against e.g., mice and rats) is therefore essential. Pets should not be allowed in and in the vicinity of the layer houses. *Dermanyssus gallinae* can also survive on cadavers (46), so these should be removed as fast as possible, at least daily, and preferably transported in sealed bags or clean buckets. A cadaver storage room in the vicinity of the layer houses or against the outside wall of the laying hen facility should be avoided and it should be regularly cleaned and disinfected after removal of the cadavers from the premises.

The infrastructure and nearby environment of the poultry farm are factors which may contribute to reinfection of *D. gallinae* and increased population growth. As storage of manure is also a source of (re)infection, it is best to have it far from the layer houses. *Dermanyssus gallinae* thrives in environments with a lot of crevices so it is therefore important to have all cracks and crevices tightly sealed and open tubes are to be avoided. Smooth materials and environments do not favor mite proliferation. In multiple housing systems, separate tools in different houses should be used to prevent dispersion. All infrastructure that is not easy to clean should be avoided.

### Suppressive Measures

During the production cycle, several actions or treatments can be applied to prevent the present mite population from growing to significant numbers. Good hygiene measures have been shown to have such suppressive effects on the mite populations (33, 47). One of the most important actions in housing systems with manure belts (enriched cages and multi-tier aviary systems) is the regular removal of manure. A test at 20 commercial layer houses (41) showed that removing manure more frequently (six times a week instead of 1–2 times a week) resulted in a significantly reduced relative growth rate of the *D. gallinae* population per house, with a higher reduction seen with higher number of mites (−79% reduced growth) of the initial population when compared to the reduction of a lower number of mites of the initial population (−53%). Cleaning the manure belts could also help, certainly if the same manure belt goes through multiple houses. Some other management actions applicable in all housing systems and enabling control of *D. gallinae* are removing dust, manure, and egg debris accumulations regularly.

Control means that can be used proactively to suppress the population growth during a production cycle are: an electrified perch (Q-perch®, to be installed before the arrival of the flock), predatory mites, and repeated treatments with inert substances (e.g., silica). Plant-based feed additives can be administered to sustain the health of the hens. Other treatments are still in development and might also be used as preventive measures in the future: vaccines and entomopathogenic fungi. All possible treatments, established or in development, are discussed more in detail in further sections.

## Monitoring Population (Step 2)

### Monitoring Tools

An essential step in IPM strategies against *D. gallinae* is adequate monitoring of the population. As *D. gallinae* is an ectoparasite which does not live on its host, the size of the population is not easily estimated. Furthermore, *D. gallinae* hides in cracks and crevices, thus the major part of the population is mostly hidden (23). Monitoring of *D. gallinae* is, to date, not often implemented in commercial layer farms. However, without proper monitoring tools, mite infestations are only noted when aggregations are visible, when there are blood spots on the eggs and/or when workers perceive irritation or bites. When these signs appear, the infestation is already rather heavy, and it is usually too late for a successful control (19, 48). Monitoring techniques should be able to determine population dynamics and spatial distribution; detect low numbers of *D. gallinae*; determine the effect of treatments; provide knowledge about the population on-farm; detect when a critical threshold for treatment has been reached; and should be species-specific (49, 50). Various different tools have been developed to monitor populations of *D. gallinae* in layer houses (Table 3). The most popular, commonly used, or promising methods are discussed in detail below.

**Table 3:** List of main techniques for monitoring poultry red mites [adapted from (23) and (50)].

**Monitoring method**	**References**
ADAS© Mite Monitor	(ADAS Ltd, Oxon, UK)
Perch trap	(51)
PVC pipe with 13 holes and towel sheet inside	(52)
Tube containing fabric or cloth	(33)
Corrugated cardboard trap	(53)
Tube trap with a wooden stick (Rick Stick) or corrugated cardboard (Avivet)	(54)
Detecting PRM in dust feathers and impurities	(55)
Examining dried droppings for PRM presence	(56)
Folded paper	(56)
Visual Mite Monitoring Score (MMS)	(57)
Velcro band mite trap (MTT)	(58)
Lohmann Trap	(59)
Modified trap after Safrit and Arends	(60)
Semi-Attractive Trap (SAT)	(48)
Simplified Passive Trap (SPT)	(61)
Scout box app	Cropwatch BV
Automated Mite Counter	(23)
Q perch counter (spinn-off from the Q-perch)	(62)
Paper tube trap	(63)
Plastic containers with heating pads	(64)
AviVet trap	(50)

In the Mite Monitoring Score (MMS) (57), no mite traps are used. Here, a visual perception of the mite infestation is performed on different points at different levels in the layer houses. At each point, an area of 1 m^2^ is observed and a score of 0–4 is given to estimate the infestation rate.

In other techniques, mites are also assessed visually but tools are used to trap the mites and score the mites in/on these traps. With the Rickstick method (54), a 12 cm long wooden stick is placed into a 10 cm long PVC tube which is attached under the perch. For scoring the infestation, the wooden stick is pulled out of the tube and the number of mites is visually assessed with reference images, also with a score of 0–4. With the semi-passive trap (SPT) (48, 61), mites are scored visually after trapping them with a piece of adhesive tape. A 4-state rating (Score 0–3) and two 2-state ratings (mites absent/present or <10 mites/>10 mites) have been proposed.

In some other monitoring techniques mite numbers are assessed quantitatively, mostly also with the use of traps. Corrugated cardboard traps, as developed by Nordenfors and Chirico (65), are one of the most popular. Here, corrugated cardboard is put inside plastic/PVC tubes that are attached in the housing system (50, 54). Before counting, the traps are usually collected in sealed plastic bags and frozen at −18 to −20°C for at least 2 days to kill the mites. To count the mites, they are all collected from the plastic bag, trap, and dismantled cardboard in a Petri dish (50, 65). After collection, the mites can be counted either manually or with computer-based estimations [e.g., using the program ImageJ, (66)] after taking a photograph (50). Cardboard traps can also be impregnated with acaricides or non-chemical control means like plant-based products or entomopathogenic fungi (see further) to use them as a control mechanism instead of solely as a monitoring tool [(67–69)].

Schulz (60) developed a monitoring trap consisting of a blood test tube filled with corrugated cardboard and compared her trap with the visual MMS method at 16 houses. With the method of Schulz (60), the presence of *D. gallinae* in the layer house was detected earlier than with the MMS method. Moreover, only one of the three mite-free houses according to the MMS method was also mite-free according to the method of Schulz (60). No correlation was found between the MMS score and the number mites per trap.

Mites can communicate with each other (70), signaling others that they have found a suitable hiding space, i.e., the cardboard trap. Therefore, the number of mites in those traps does not correspond accurately with the number of mites effectively present at the place of the trap. This issue is resolved in the use of Semi Attractive Traps (SAT) (48), where mites are attracted to traps with water. Here, small plastic jars with perforated lids and filled with water containing 0.01% surfactant are placed in the layer houses. When the mites enter the jar they are drowned by the water and surfactant and thus cannot communicate with each other. The number of mites in those traps are more representative of the mites actually occurring at that place.

Most monitoring methods or tools are not validated, only tested under practical circumstances. Recently, the AviVet trap, which uses the principle of corrugated cardboard within a barcode-labeled Tylene tube (for identification), has been validated (50). The process of collecting mites is similar to that described above for the regular cardboard traps, but the mites are weighed instead of counted. The validation process indicated that the weight correlates for 99.6% with the counted number of all stages, under the following regression line: Y = 58.8 + 9.56x, where Y = total number of mites in the AviVet trap and x = the weight of all mites in the AviVet trap. In addition, the trap was proven to be *D. gallinae*- specific (50).

As all of the above mentioned monitoring techniques are quite labor intensive, an ‘automated mite counter’ has been developed and validated (23, 49). This counter is placed under the perch. Mites enter the counter through a hole in the lid (again mimicking a hiding space), and are detected and counted by a sensor device. When detected, the mites are removed by air suction into a filter. The mites stay in this filter until removal, which should be done weekly to prevent blockage of the system (23). Validation confirmed the efficacy of the automated mite counter, providing a good estimate of the number of mites, even at low infestation levels. Moreover, the counter seems to be species specific, only counting *D. gallinae* and no other mite species (23). The device is currently commercialized for use in laying facilities as MiteAlert® (49). In this commercial counter the mites are not collected in the filter but blown out by the pump back into the hen house.

Not all of the monitoring methods mentioned above are suitable for routinely sampling by the farmers themselves. Some studies have revealed that farmers favor methods that are not too labor intensive, like the Rickstick method (54), the Simplified Passive Tape (SPT) trap (61), the AviVet trap (50) and the Velcro trap (58) in which a section of Velcro wrapped around the perch provides a hiding place for mites (41, 71). The automated mite counter (23) is also one of the preferred methods of farmers as it is a practical tool for monitoring with minimal workload.

### Location, Frequency, and Duration

The location of the mite traps in the layer houses is crucial for correct monitoring. They should not be placed in mite aggregates, but in passages frequently used by the mites. Near the perches or at cross-points are well-suited localities (50). Traps in nest boxes are less accurate, probably because hens spend less time there, certainly not at night when the mites become active (56). To follow-up the mite population over time in the same poultry house, traps should be placed at exactly the same site on each occasion. Therefore, a detailed map of the trap locations in the layer houses is very useful (50). The number of mites counted with the traps may not be representative of the actual number of mites in the layer houses, but they allow actions to be taken following a change in population size.

A recent project (41) analyzed a large dataset of mite monitoring data to identify the ideal number of traps and trap distribution for obtaining a good insight of the true infestation level. The results revealed that the more traps are placed, the better the insight into the actual infestation level and a minimum of 12 traps per house should be placed. Further, the distribution of mites between and within houses and between different flocks proved to be very variable and thus unpredictable. Therefore, an even distribution of traps over the houses should be applied.

Concerning the frequency of monitoring, again the more frequently monitoring is performed, the better insight is acquired into the population dynamics (72). However, in practice, a compromise should be found with the workload of the monitoring for the farmer. Recommendations by the COST action “COREMI” (FA1404), advise routinely 2-weekly monitoring during commercial production, and weekly in the framework of an experimental design. Although monitoring weekly or 2-weekly, all traps using corrugated cardboard should only be placed in the layer house for about 3 days before removal, as those traps reach a peak of collecting capacity on day 2, and traps that are placed for a longer period are often more damaged by the hens ((65)). Some traps like the Semi Attractive Traps (SAT) (48) and the Rick Stick method (54), can remain for 2 weeks in the layer houses.

The monitoring of mites in layer houses can still be fine-tuned. Quantitative monitoring methods seem more informative than scoring techniques (i.e., no mites- very many mites) (73). However, mites are spread heterogeneously and often unpredictably in a layer house, making it virtually impossible with currently available methods to assess the actual infestation at time “t” with traps. A very high number of traps would be needed to approximate the general infestation level in the house. Also, as mites attract each other to the traps, the number of mites in most traps do not correspond accurately with the number of mites effectively present at the place of the trap (see above). Moreover, comparing actual numbers of mites between houses is difficult as they depend on a large range of different factors (23). Temporal evolution of the population growth within a layer house is thus more relevant (23, 48). The automated mite counter (23) is a method that works with population growth instead of actual numbers.

Actually, the use of simple binary traps might be promising to get relevant information. Chiron et al. (48) already illustrated a very high correlation between a more exhaustive notation of mite aggregates (e.g., four-state scoring) and SPT binary scores. Binary scores are much less labor intensive than multiple-state scoring, and certainly than counting the actual number of mites. Therefore, it would in practice allow the assessment of more traps. As population growth is more relevant to assess the problem (48), and is also used in studies to determine a threshold (see below), the use of a relatively high number of easy-assessable binary methods might the appropriate methodology for use within an IPM strategy.

## Treatment Decision Based on Monitoring and Thresholds (Step 3)

Preventive actions alone are often not sufficient to fully control the pest, and curative means often need to be implemented. Even then, complete eradication of *D. gallinae* is virtually impossible, and control measures should instead be aimed to keep the infestation under a so-called economic threshold, to avoid negative effects on the hens, humans and production. A critical point in an IPM strategy is timing actions (e.g., altering preventative measures or adding treatments) to prevent the increasing pest population from causing damage. By using this “action threshold,” treatment/action is not performed too soon and too much, avoiding negative effects on the environment, redundant costs, and resistance emergence. Treatment is also not performed too late so efficient control is still possible.

Unfortunately, such general thresholds are not yet available for controlling *D. gallinae*. This lack of thresholds largely hampers the development of generally applicable IPM strategies for layer houses, and further research is urgently needed. Several monitoring techniques or treatments (see further) provide their own thresholds where they advise treatment is necessary, though these are not scientifically proven.

The difficulty in determining thresholds lies partly in the complexity of determining the actual population size and the multitude of influencing variables (see above). In addition, although production losses due to *D. gallinae* infestations have been evidenced (16), the exact relationships between infestations and bird health/economic impact are insufficiently known. Also here, the many influencing factors hamper the determination of economic thresholds.

A first promising study toward a general critical action threshold was based on concomitant SAT and SPT trapping (48), and working with the temporal evolution of the trapped *D. gallinae* population size. This method is based on Verhulst's mathematical model, which describes the temporal dynamics of populations of living organisms in three successive phases (latency phase, exponential growth phase, equilibrium phase). To allow farmers to treat before the population growth phase, they proposed (1°) characterize the temporal dynamics of mites by monitoring from the beginning of the flock over several months in order to capture the latency phase and the growth phase by trapping both with a fairly accurate tool and with an any easy-to-use tool in parallel (here SAT and SPT resp.); (2°) identify the moment of maximum acceleration by fitting the Verhulst model to the most accurate data (using a solver tool); (3°) position this key moment on the data obtained concomitantly with the easy-to-use tool and see whether there's a recurring change before he does. Chiron et al. (48) observed in three commercial farms that when for three successive monitoring moments >20% of the placed SPT contained mites, this key moment was approached. Such an SPT-based threshold can easily be applied by the farmer himself. This opens up interesting perspectives for defining a critical threshold that can be associated with monitoring by any means (SPT, Ricksticks, Velcro traps, automated counter, Avivet.). Research on this threshold is still ongoing and it needs further refinement before it can be used with confidence. Although the SPT-based threshold prevents the mite population to start growing exponentially, implicitly avoiding the point of economic damage, it does not explicitly take this economic impact into account.

Another prospect toward treatment advice is the dynamic adaptive model (DAP) (72). The DAP model can predict the population dynamics of *D. gallinae* in a layer house based on monitoring data of the current flock population, temperature data and treatment dates. The model deals with variation in population dynamics by including flock-specific parameters. The model could, however, still be improved by e.g., including more parameters like flock age or husbandry measures. Further research on the DAP model (41, 72) was executed at three different laying hen houses: organic aviary, aviary with winter garden and aviary without winter garden. This research resulted in a practical applicable model forecasting the mite population growth. At the same time, an economic model and an advice algorithm was tested at these farms in order to determine the optimum interval between two treatments in order to keep the population under a certain growth level and to determine if an extra treatment is cost-effective by getting lower numbers of mites and therefore better feed conversion. The threshold and moment when exceeding the threshold was different for all layer houses as e.g., cost price of the eggs, treatment costs and treatment efficacy was different for all layer houses. This illustrates the difficulty of determining general applicable action and economic thresholds.

## Non-Chemical Treatment- Methods (Step 4)

In IPM, the use of chemical (synthetic) control strategies is as much as possible avoided in order to reduce negative effects on human and animal health and on the environment. During the last decades, a lot of effort has been, and is still being, devoted to investigating alternative control measures. The current state-of-the art of alternative control mechanisms against *D. gallinae* is listed below and summarized in Table 4.

**Table 4:** Overview of main non-chemical treatments.

**Treatment**	**Mode of action**	**+**	**–**	**P/C**	**Comm**.	**References**
**Plant-derived products**	Acaricidal, toxic	Short environmental persistence	Short effect	C	X	(74–76)
	Repellent	Potential in attract-and-kill	Lack of standardization	P	X	(77)
**Vaccines**	Boost immunity	-Low risk for resistance -No workload during production	Further research needed for commercialization	P		(78)
**Biological control**						
*Predatory mites*	Prey on PRM	No negative effect on environment (natural enemies)	Also affected by other treatments (silica, acaricides,.)	P(/C)	X	(79, 80)
*Entomopathogenic fungi*	Penetrate host	Potential in traps	Suboptimal conditions in layer houses	C	X	(81)
*Nematodes + endosymbionts*			Much research still needed			(82, 83)
**Physical control**						
*Inert dusts (on system)*	Dessication of PRM	-Resistance less likely (mainly physical mode of action)	-Health hazards (esp. crystalline) -Variability in effectiveness	P/C	X	(84, 85)
*Q perch*	Electrify PRM	-No harm to hens-Resistance less likely	Expensive, change in infrastructure	P	X	(62)

*Treatments non-allowed in EU (light regime, oils) are not included. Main advantages (+) and disadvantages (–) are listed, as well as their use (P, preventively; C, curatively) and whether they are commercially available (Comm.). For each treatment, main references are given. For further details and other references: see text*.

Although the non-chemical treatments are here listed under step 4, meaning curatively after the mite population has exceeded a threshold, they can within IPM strategies, also be used preventively (step 1) to suppress the growth of the red mite population.

### Plant-Derived Products

Plant-derived products have promising potential as alternative non-chemical methods against *D. gallinae*. They can have acaricidal, toxic activity, but also repellent or attractive effects on *D. gallinae* (75, 86, 87). The repellent and attractive effects have much potential in combination with other treatments and are therefore discussed in detail in the section ‘treatment combinations’. As plant-based products mostly have low toxic effects on mammals, and are said to have a short environmental persistence, they could have a rather low impact on the environment (74, 75, 77). To act as a repellent, even lower dosages are required then to act as a toxicant (88). These characteristics make plant-derived products very suitable for use in IPM strategies.

Several studies demonstrated that the efficacy of essential oils against *D. gallinae* is mainly attributed to effects of their volatile components (75, 89–91), indicating that they probably have a neurotoxic effect rather than a mechanical one, which indeed has been demonstrated by López and Pascual-Villalobos (92).

The fact that essential oils mainly act through their vapor phase is on the one hand an advantage, as in that way the mites in hiding places can also be reached. On the other hand, the volatile nature probably is the cause of the rather short effect of many oils (88). Another major limitation of the use of plant essential oils is the lack of standardization in formulation, with different batches having differences in chemical composition, which can result in inconsistencies in acaricidal efficacy (75, 88). This problem might be overcome by isolating the active compound of the essential oils, of which eugenol currently seems to be promising, showing the highest toxic effect against *D. gallinae* in several studies (91, 93). In the latter, eugenol also showed to have a repellent effect, changing to an attractive effect over time. Additional research on these active compounds is still necessary.

Further, the efficacy of the majority of these plant-based products has only been demonstrated at lab scale, while in the field, efficacy might be affected by environmental factors like humidity, dust and other pesticides used (91, 94). Also, although some essential oils do not show any negative effect on hens (e.g., thyme), others (e.g., pennyroyal) appear to have an impact on chicken health and egg production (76). Finally, given their short residual toxic effect, plant products may not be suitable as a stand-alone treatment against *D. gallinae*. As such, it has been suggested to combine plant-derived acaricides with other treatments with longer-term effects for a more efficient control of *D. gallinae* (94). Plant-based acaricides are meant to be used curatively, thus ideally after passing an action threshold.

Some plant-based acaricides are commercially available (although not allowed in all countries) for use in layer houses. An example is MiteStop® (Alpha-Biocare GmbH), a product based on neem seed extract (*Azadirachta indica)*, which has proven its efficacy against *D. gallinae in vitro* as well as in field conditions (95–97).

### Vaccines

With vaccination of hens, there is a low risk of environmental contamination, and the risk for resistance emergence in mites is highly unlikely (98). As such, vaccines have much potential to be incorporated in IPM for *D. gallinae*, principally as a method of prophylaxis as mass administration by injection is not possible once the hens have entered the layer house. Nevertheless, once suitable vaccine antigens have been identified, methods to boost immunity or to administer therapeutic vaccines through e.g., the drinking water during the laying cycle should be explored.

During an infestation, some antigens of the parasite are continuously exposed to the host. These are known as “exposed antigens.” In the case of *D. gallinae*, however, the natural immune reaction of the hens against these “exposed antigens” does not seem to be effective for controlling the mites (98, 99). Therefore, the focus for developing vaccines against *D. gallinae* lies on the use of “concealed antigens,” i.e., antigens that are not exposed to the host during blood-sucking, like some proteins associated with the midgut of *D. gallinae* (100). This strategy, of using a concealed midgut-related antigen, has previously led to the development of an effective vaccine, marketed as TickGARD, against the cattle tick *Rhipicephalus microplus* based on the BM86 protein (98).

For *D. gallinae* the development of vaccines, including the search for candidate antigens, is still an ongoing process, which has gained momentum since the upsurge of genomics and the recent publication of transcriptomes and the genome of *D. gallinae* (101, 102). Several studies have demonstrated the potential of both native (autogenous) and recombinant antigens for vaccination against *D. gallinae* (78, 98, 99, 103). In an autogenous vaccine, mites for antigen extraction are used from the same premises for which the vaccine will be used. Although authorization for the production and use of these kinds of vaccines may be more easily obtained than for defined vaccines produced on a commercial scale, producing autogenous vaccines is very labor intensive, as mites from every premises that will be treated need to be sampled to make a farm-specific vaccine. Furthermore, in autogenous vaccines, the effective antigen(s) are not well-defined and, as such, poorly quantifiable. Therefore, the efficacy of autogenous vaccines cannot be quantified and may vary between batches. In spite of these difficulties, in a recent field evaluation of both an autogenous vaccine and a prototype recombinant vaccine (78), the autogenous vaccine led to a reduction of 78% of the mite population, while the recombinant vaccine did not show a significant difference in mite numbers compared to the control group. While an autogenous vaccine is therefore currently a possibility for use against *D. gallinae* on a small scale, developing an optimal, standardized recombinant sub-unit vaccine with prolonged efficacy to avoid the need for boosting is the long-term goal (78).

### Biological Control

The use of natural enemies against *D. gallinae* is well-framed within IPM strategies, and already commonly applied in horticulture. By using biological control measures, the impact on the environment is minimal and the risk of resistance emergence is avoided.

#### Predatory Mites

Naturally-occurring enemies can be of great benefit to control pest species. For *D. gallinae*, being an ectoparasite, using enemies that share his living environment is promising (80, 104). The natural communities of enemies of *D. gallinae* occurring in the layer house should be valued, as these already can (partly) control the poultry red mites. Some natural enemies are also artificially reared and commercialized, to mass release them for controlling pest species. This methodology is certainly effective in enclosed systems where the natural enemies are confined to the release site (21). As such, this strategy is already widely applied in greenhouses (105). Candidate predators for commercialization (mass releases) to control *D. gallinae* have been identified by searching for natural occurring predator mites in poultry houses and wild bird nests. *Androlaelaps casalis, Cheyletus eruditus, Hypoaspis aculeifer*, and *Stratiolaelaps scimitus* (previously *H. miles*) are identified as genuine predators of *D. gallinae* (79). As these species naturally occur in layer houses, the risk of a substantial impact on non-target biodiversity is limited, although it remains to be tested. These predators are not *D. gallinae*-specific, also predating on other arthropods and even among predators (80).

*Hypoapsis* species have a very high predation capacity against *D. gallinae*, higher than that of *A. casalis*, but they appear to be less mobile (106). In addition, for *Hypoapsis* spp., it seems to be impossible to obtain an established population in poultry houses, as specimens were never retrieved a week after release (Koppert Biological Systems).

At least in Europe, currently mainly *A. casalis* and *C. eruditus* are introduced as predators in commercial layer houses, under their respective commercial names ANDROLIS® and TAURRUS® (Koppert Biological Systems, the Netherlands). ANDROLIS® and TAURRUS® can be used complementarily. ANDROLIS mainly feeds on juvenile stages of *D. gallinae* and is a highly mobile and active hunter, while TAURRUS preys on all stages of *D. gallinae* and is a wait-and-sit predator, with a rather slow dispersion. ANDROLIS prefers more humid microhabitats, while TAURRUS prefers drier places (nests, perches,.). Predatory mites are usually released preventively, with the protocol of numbers, frequency and locations of releases depending on the number of hens and housing system. When the populations of *D. gallinae* become too high nevertheless, extra releases of both species can also be done curatively.

The use of predatory mites are promising as a part of a combination of treatments within the IPM strategy, although it should be taken into account that other treatments for *D. gallinae*, like synthetic chemical, plant-based acaricides, or inert substances, also can have deleterious effects on the (natural) predators of *D. gallinae* (80).

#### Entomopathogenic Fungi, Nematodes, and Bacterial Endosymbionts

There is potential for entomopathogenic fungi, nematodes and bacterial endosymbionts as non-chemical control measures against *D. gallinae*. However, studies on their use against *D. gallinae* in poultry houses are not advanced enough yet, there are still too many hurdles for them to be effectively used in practice, and they are not yet commercially available for use against *D. gallinae*.

Entomopathogenic fungi are frequently used, worldwide but mainly in Latin America, to control pests in crops (21, 81). Fungi infect insects and mites with their spores that adhere to the hosts' cuticle, germinate, and penetrate and spread into the hosts' body (81). Studies under lab-scale conditions have shown that *D. gallinae* is specifically susceptible to *Beauveria bassiana, Metarhizium anisopliae, Trichoderma album*, and *Paecilomyces fumosoroseas* (107–112). However, some experiments in semi-commercial conditions show these fungi are unsatisfactory as a control mechanism against *D. gallinae* (113). In addition, humidity levels in poultry houses are too low to ensure fungal transmission (21); and the effectiveness of these fungi also highly depend on the fungal strain (110). Further, as these fungi are not selective for *D. gallinae* only, they may negatively affect the environment, leading to an environmental disequilibrium (100). However, they are harmless for poultry, eggs and humans (112). These fungi seem to have most potential for being used within traps placed in the poultry house (81), for example within an attract-and-kill strategy (see below). Indeed, a recent study of Nascimento et al. (68), demonstrated the successful use of *Beauveria bassania* in autoinoculation devices (traps with corrugated cardboard or loofah sponge) to control *D. gallinae* in both lab conditions and poultry houses. With this mode of application, problems with non-optimal environmental conditions in poultry houses for the fungi are overcome.

For nematodes, no field experiments on their efficacy against *D. gallinae* have been carried out yet, but using them for controlling flies and beetles in poultry houses has been ineffective, despite positive results in lab-conditions (82). Both fungi and nematodes require particular environmental conditions, such as high humidity levels and free water, making it very hard to use them in practice in poultry houses (8, 21).

Targeting endosymbionts that are vital for mite reproduction and growth is another pathway that is being investigated for control of *D. gallinae*. Although some endosymbionts have been identified in *D. gallinae* with DNA sequencing (83), further research is certainly necessary before it can be used in practice as a treatment.

### Physical Control Mechanisms

Hygiene measures, cleaning and disinfection can also be regarded as physical control and these have been discussed in the “prevention” section. Also heat treatment, which can only be applied during the empty period, is discussed in that section.

#### Light Regime

Studies have shown that mite populations can be affected by disrupting light-dark cycles. Host-searching activity of *D. gallinae* starts during the dark period, with the highest activity 5–11 h after darkness, so when this dark period is interrupted, their host-seeking activity will decrease and their natural feeding cycle will be disrupted (114, 115). Indeed, the reduction of numbers of *D. gallinae* by short-cycle intermittent light/dark periods has been illustrated by Zoons (116) and Stafford et al. (117), and prolonged darkness compared to standard light regimes resulted in increased number of mites (115). However, disturbing the dark-light rhythm will also largely affect hens, and EU legislation requires a statutory dark period of 8 h (1999/74/EC), so this technique cannot be applied as such in Europe.

#### Inert Dusts

Inert dusts, or silica-based products, contain *silicon* dioxide as the active biocidal substance, which is, at the time of writing, one of the few permitted biocidal products allowed for treatment of *D. gallinae*. Both synthetic and natural silicas exist. Synthetic products contain only amorphous silicon dioxide; natural products are mainly based on diatomaceous earth and contain a small amount (<1%) of crystalline silicon dioxide. In several regions, only natural silicas are allowed for use at organic farms. However, the amorphous form is considered relatively healthy, while the crystalline form in natural silicas is more harmful to environmental, animal and human health (85, 118). Synthetic silicas are thus actually not truly non-chemical treatments. However, their mode of action against *D. gallinae* is said to be completely mechanical (not poisonous), drying out the epicuticle of the exoskeleton of the poultry red mites through the absorbent character of the silicon dioxide particles, leading to desiccation of the mites (84, 119). Furthermore, resistance against this physical mode of action is less likely to develop than to single-target molecules ((85)).

Many of these silica-based products are commercially available and widely used in Europe. Since dusts consist of fine particles, they can pose hazardous effects to the respiratory tracts of humans. Therefore, a shift is noticeable toward the use of liquid silica-based products, to reduce the deleterious effects of airborne silica (30). However, these effects are not completely eliminated with the liquid application of silica, as some particles become airborne when the fluid has dried, and are further dispersed by hens. *In vitro*, diatomaceous earth seems more effective than synthetic silica (30), though the latter seems to have a longer effect in the field in its liquid form compared to diatomaceous earth (85, 120). The effectiveness against *D. gallinae* has been proven to vary between different silica-based products (especially amorphous silicas) due to variation in absorption capacity of particles, chemical composition, particle size, and specific surface (85). In addition, the effectiveness of inert dusts greatly diminishes at humidity levels of >85% (which can easily be reached in layer houses) (30, 84). Inert dusts are often used preventively in-between flocks, though repeated applications during production are often necessary (84). Even with repeated treatments, silica is often not sufficient as a stand-alone product to control infestations of *D. gallinae*. It is observed that the effect of silica declines after repeated treatments, which could be related to flock age or to the accumulation of dust and debris in laying hen houses over time, hampering the efficacy of silica (72). The latter could be avoided by mechanical cleaning of the surfaces prior to silica application, which has indeed proven to increase the efficacy of silica (120).

#### Oils

*in vitro* studies illustrated the effective use of the spraying of mineral oils, diesel oil, petroleum, and plant oils (rapeseed and concentrated orange oil) for poultry red mite control (30). These oils have mainly a physical action by obscuring the stigmata, preventing normal breathing of the mite (106). With diesel oil, there is an associated risk of egg contamination (30). A large disadvantage of oils, is that the greasy substance can stain the eggs, affect the functioning of parts of the system like the egg belt, and they are also rather difficult to remove by cleaning. Further, if not applied everywhere, mites can just avoid the oil spots. Different oils have been used by farmers for years, often successfully controlling mite infestations. However, these oils are not currently permitted for use as a treatment against *D. gallinae*, as there is no biocide registration for any of such products.

#### Q Perch

The Q perch® (Vencomatic, Netherlands) is a perch where two electric wires and insulators are installed under the cap of the mushroom-shaped perch. Due to the design, the hens cannot be harmed. A small electrical current runs through the wires, killing the mites on their way from their hiding place to the hens nightly resting place (62). The efficacy of the Q-perch is not demonstrated in scientific literature, and installing a Q-perch is rather expensive and a pervasive change of the infrastructure. As it needs to be installed in the layer house, it is used as a preventive measure to control mite population growth, not as a curative measure.

### Treatment Combinations

As researchers are becoming more aware that current control mechanisms on their own are not sufficient for controlling *D. gallinae* in layer houses, studies are being performed examining the added value of combining multiple treatments as some can work additively or even synergistically. Sparagano et al. (22) proposed a predicted compatibility matrix of existing and emerging control strategies (their Table 1). However, as only few combinations have actually been scientifically tested, that matrix should be mainly seen as a prediction of compatibilities based on the mode of actions of each treatment separately.

One of the few combinations that has been scientifically demonstrated to have beneficial effects is the combination of the fungus *Beauveria bassania* and inert substances (desiccant dusts) (121). Although this study only comprised laboratory experiments, and the combination still needs to be tested on the field, it illustrates the potential added value of combining treatments.

Two consecutive projects demonstrated the potential of combining predatory mites (*A. casalis* and *C. eruditis*) with two acaricides (milbemectin and amitraz) using impregnated cardboard traps. The test in actual layer houses (cage and aviary systems) illustrated that the combinations had a better efficacy than all the treatments separately. However, the combinations were still not sufficient to control high *D. gallinae* infestations. “Triple” treatments, where plant-based products were also added, had higher efficacy [(45, 69, 106)].

Like some plant insect pests, *D. gallinae* must find its host at a distance since it does not live on it. *Dermanyssus gallinae* is known to be attracted to temperature gradients and to CO_2_ puffs, which are features of homeothermal vertebrates that can be detected at a distance, but are not host-specific. Interestingly, CO_2_ is attractive in the dark but induces the mite to freeze in the light, a behavior which apparently allows *D. gallinae* to avoid being eaten by its host (122). *Dermanyssus gallinae* is also attracted by host-specific pheromones, emitted by the hens (123). A cocktail of five volatile compounds among the dozens of compounds naturally emitted by hens has been patented for its attraction demonstrated under controlled conditions in the laboratory (124). Besides host-related attractants, *D. gallinae* is also attracted by aggregation pheromones of congeners, causing clustering together (70, 125). Though the pheromone has not been fully characterized chemically, a series of compounds isolated from the odor of freshly fed *D. gallinae* has been patented as attractants (126).

Interfering in the sensory interactions between *D. gallinae* and its host or between mites among each other are promising avenues to progress IPM, and such approaches may also have minimal environmental impact. The automated mite counter (23) was designed to attract mites as it provides a heat source. Some plant-derived products also act on the sensors of *D. gallinae* as they have a repellent or attractive effect on *D. gallinae* (see above). Attractants and repellents have much potential for use in certain combinations.

The so-called “attract-and-kill” and/or “push-pull” techniques have an interesting potential for a mite that does not live on the host. The principle of the attract-and-kill (or lure-and-kill) technique, widely deployed in crops [e.g., (127)], consists in diverting the pest from its target (host/congeners) by competing odors to specific sites equipped to kill it by various means like acaricides or entomopathogenic fungi. In the case of *D. gallinae*, mite traps (as used for monitoring) impregnated with eradicating treatments are used [e.g., (67)]. Within an IPM strategy, non-chemical killing agents are preferred (24). By using pesticides only on traps, the total amount of pesticide used is also reduced, resulting in less impact on the environment. Further, resistance emergence is slowed down with this methodology as the parasites are exposed to effective doses in a contained environment (70). However, the effective implementation of an attractive substance in such a method has many impediments. The attractant must overrule the natural attractants (host/congeners) interacting with sensory system of *D. gallinae;* an efficient slow-release system for the attractant must thus be developed; and the olfactory attraction must be important in the behavioral choices of *D. gallinae*. The push-pull method combines a repellent activity to push the pest away from its target and attract it to another element. This way the attract-and-kill strategy is actually enhanced by adding a repellent into the process. To our knowledge, however, it has never been used to combat *D. gallinae* to date. Repellents alone can also be used, e.g., by preventing *D. gallinae* to hide into the cracks and crevices, resulting in *D. gallinae* becoming more reachable with (preferably non-chemical) contact products.

Certain plant-based food or drinking water supplements exist for increasing or sustaining the hens health and natural resistance, which also have an effect on the odor of the hen, rendering it less attractive for the mites, and as such working as a repellent (123). This can be beneficial as mites will feed less, and starved mites seem to be more susceptible to acaricides and possibly also to desiccants (128). The potential of combining these supplements with other treatments, i.e., predatory mites and acaricide-impregnated traps, has been demonstrated in field conditions [(45, 69)].

Not all treatments can be used in combination, and some probably have antagonistic effects. Broad-spectrum approaches like silica or heat treatment, e.g., might have adverse effect on the use of natural enemies. Besides combining actual treatments, treatments can easily be combined with simple management actions like cleaning places where hotspots are found with water and soap, to keep the infestation under control, which has proven to be effective (8, 19, 41). The influence of different housing systems and of parameters like temperature and humidity on the efficacy of any treatment (combination) has also been illustrated [e.g., (45)]. Using (or building) systems that are less beneficial for poultry red mites, and limiting the number of potential mite refugia could also help controlling infestations (22). All this illustrates that a holistic approach; integrating biosecurity and prevention measures; appropriate monitoring; attention to different conditions in different housing systems; and interactions with environmental conditions, will be indispensable for effectively controlling poultry red mites in layer houses.

## Use of Selective/Specific Synthetic Pesticides (Step 5)

In IPM, the use of synthetic chemical treatments is not completely ruled out. The idea is to only use chemical synthetic products as a last resort, when non-chemical synthetic treatments proved not to be sufficient to control the pest species (24). It is important to thoughtfully select the acaricide and to minimize the total quantity of chemical product that is applied, though respecting the advised dosages to avoid resistance emergence in the pest and optimize the chances of success of the treatment. Preferably, selective products that only affect the target species (*D. gallinae* in our case) should be applied. This way, side effects on non-target species are avoided and the impact on animals, humans and the environment in general is reduced. Unfortunately, at the time of writing, none of the chemical synthetic products available are absolutely selective for *D. gallinae*, as they also have toxic effects on other insects and arachnids.

It is very difficult to obtain information on which synthetic chemical acaricide is currently allowed for use against *D. gallinae*, and different regulations exist for products classified as biocides or veterinary medicines. The products allowed in Europe can be found on the database of the European Medicine Agency database (www.ema.europa.eu) for veterinary medicines, and on the European Chemical Agency database (www.echa.europa.eu) for biocides. These lists are, however, rather user unfriendly, and farmers request more transparency. Furthermore, they are dynamic and can change daily, making it necessary to regularly check for allowed products. Currently, every EU country has its own national admissions, which makes it even more confusing which product is licensed in a specific country. At the time of writing (2020), only the organophasphase phoxim (ByeMite®), spinosad (Elector®), fluralaner (Exzolt®) and some products of silica/diatomaceous earth are allowed in most European countries (although national regulations should be checked). All these products are allowed during production, although with some restrictions.

The organophosphate phoxim (ByeMite®) is licensed as a veterinary medicine against ectoparasites for livestock (including layer hens), but is not allowed for organic farming. Although some studies report high efficacy of ByeMite® in multiple systems after repeated application (129, 130), others report variable results among different regions and different contact duration (96). In addition, resistance emergence against phoxim is already reported in several countries (see below).

Spinosad (Elector®) is a biological acaricide, and acts upon the nervous system of the mite. Spinosad is licensed as a biocide, and is also allowed for organic farming. Tests in actual layer houses, however, illustrated that the effect of Elector® does not last long and that it is not sufficient as stand-alone treatment (106).

Recently, a fluralaner-based product called Exzolt® has been marketed, which is the first systemic acaricide against *D. gallinae* that is administered orally through the drinking water. It is classified and licensed as a veterinary medicine. It is also allowed in organic farming, though with an extended withdrawal period. Fluralaner is an isoxazoline and inhibits the ligand-gated chloride channels, with a high selectivity for the nervous system of mites, thicks and insects (131). Exzolt® has been proven to kill mites very fast, within 4 h in lab conditions (132). Also in field studies, mite reduction almost occurred immediately, and reductions of up to 100% were observed in all tested units (16, 133). The duration of efficacy (of >90%), however, varied greatly among the tested layer farms, ranging from 56 to 238 days (133).

## Reduction of Pesticide Use (Step 6)

Most chemical acaricides are applied in the form of a spray or dust, by which not all hiding mites can be properly reached. Further, spraying can cause stress in the hens; leads to an exposure of the hens and workers to the acaricide; can lead to environmental contamination through e.g., the manure used as fertilizer; and increases the risk of residues in hens and the eggs (18, 133). Indeed, studies on European farms revealed the presence of acaricide residues, even of currently unlicensed substances in tissues and organs of hens and in eggs (18, 134, 135). However, current legal limits (MRL), or MRLs in force at the time the product was allowed, were virtually never exceeded. To reduce the impact on the hens and humans, it is thus essential to reduce the amount of chemical acaricides used in the control of *D. gallinae*.

As the fluralaner-based product Exzolt® is administered through the drinking water, the amount of product that accumulates in the immediate environment is limited (133), though there is concern about the environmental impact of residues in the poultry manure if it is to be used as a fertilizer. Another strategy to restrict acaricide exposure to the environment is to use cardboard traps impregnated with acaricides instead of spraying the whole layer house (65, 67). Hanging sufficient mite traps can, however, be labor intensive, certainly in larger houses, making farmers likely to prefer spraying instead. Another alternative to spraying the whole layer house is only treating hotspots. This is successfully applied in horticulture within IPM strategies (41), and can also easily be applied in *D. gallinae* control. Hotspots with higher infestation levels, where biological treatments thus cannot control the pests, are identified with monitoring. At these restricted places, acaricides are applied with special spray devices to eradicate these populations and to avoid them spreading over the whole area. By treating locally, the total amount of pesticides used is reduced and negative impacts on natural enemies are limited (41).

## Anti-Resistance Strategies (Step 7)

Resistance against certain controlling treatments is the inheritable ability of an individual to survive this treatment. When an individual is resistant, it will not or only be little affected by the treatment. Resistant individuals will have a competitive advantage over non-resistant individuals, and will thus more likely reproduce. This way, the frequency of their genotype will augment from generation to generation in the treated population. As such, there will be natural selection for these resistant traits. After several generations of selection for resistant genotypes, there will be an evolution towards a general field resistance, and thus a large decrease in the effectiveness of that specific treatment (136). Different underlying mechanisms of resistance exist, and understanding them is crucial to optimally apply anti-resistance strategies (137, 138). For *D. gallinae*, resistance against the formerly widely applied pyrethroids is being studied, with Katsavou et al. (139) showing a high frequency and wide geographical distribution of several non-silent mutations in the voltage-gated sodium channel (the target protein of pyrethroids) associated with resistant phenotypes, though further research is needed. Bartley et al. (140) found that a glutathione S-transferase may have important roles in the detoxification of pesticides and thus in metabolic resistance. Roy et al. (141) detected variations in the enzymatic activity of acetylcholinesterase, the target of organophosphates, among PRM populations sampled in 2008–2009, perhaps foreshadowing some of the current treatment failures.

Resistance of *D. gallinae* against currently and formerly frequently-used chemical acaricides like carbamates, pyrethroids, and the organophosphate phoxim (in ByeMite®), have been widely reported (8, 18, 142). The likelihood of resistance emergence is increased when products are applied in incorrect dosages, or too frequently, which is reinforced by the limited number of allowed chemicals.

Reducing the use of synthetic chemical acaricides maybe reduces the risk of resistance emergence, but does not avoid it, all the more that resistances to non-chemical treatments are generally far less studied (143). When natural products are commonly implemented in the future, *D. gallinae* population may become resistant against these products too. To date, further research on the underlying mechanisms of resistance against non-chemical treatments is certainly necessary.

To warrant the success of IPM for controlling *D. gallinae*, actions to avoid resistance emergence against these natural as well as chemical treatments should be taken into account. First of all, to decrease the risk of resistance emergence, it is vital not to under-dose nor to exceed the recommended frequency of application of a product. Resistance emergence can be delayed by implementing certain treatments only in traps instead of in the whole system (see above). Resistance is also avoided by combining and/or rotating different products with different modes of action (138). Combining different products with different modes of action is one of the main ideas of IPM and has already been discussed in the section “Non-chemical treatments.” Also well-applying preventive management actions could highly reduce the needed amount of other or curative treatments, thus avoiding resistance emergence for those treatments.

## Evaluation (Step 8)

To assess the efficacy of an applied IPM strategy, and to determine whether adaptations are necessary, a good evaluation of the strategy is needed throughout the whole process. This is primarily done by monitoring the *D. gallinae* population continuously to evaluate the effect of the different treatments (preventive or curative, and non-chemical or chemical), and the IPM strategy as a whole. Besides info on the effectiveness of a treatment, frequent monitoring also provides insight into the duration of the effect. The latter is useful information to determine the cost-benefit of a certain strategy At the stage that IPM strategies will be implemented, the balance between efficacy and (time) costs for a strategy needs to be evaluated, also including economic benefits.

It will be virtually impossible to develop general applicable IPM strategies for every farm. Indeed, a large range of varying factors like house temperature (which is also seasonally influenced), humidity levels, husbandry systems and hen breed, between and even within farms can influence the life cycle of the mites and/or the effectiveness of the treatments applied (72). As such, even the economic and thus also action threshold may vary over farms (see above). Therefore, the aim should be to develop dynamic IPM strategies, with different options depending on specific situations. A continuous evaluation of a strategy at a certain farm at a certain time is thus essential.

## Conclusion and Future Developments

The presented overview shows that for every IPM step, elements are available for control of *D. gallinae* in layer houses. This opens new horizons for researchers in the field to develop practical IPM strategies. However, there are still some important shortcomings and knowledge gaps that hamper the delineation of practical IPM strategies for the control of *D. gallinae* in commercial layer farms.

A major knowledge gap lies in the determination of the action and economic threshold to decide at which point additional action is required. A main confounding factor is the large range of varying factors influencing population dynamics and economic consequences (see above).

In essence, most knowledge gaps lead back to an insufficient fundamental knowledge of the biology and behavior of *D. gallinae*, though some literature exists [e.g., (53, 100)]. As mentioned, improved insights into the population dynamics are essential for the thresholds determinations. Also further research on the actual effects of non-chemical treatments on *D. gallinae* is necessary. Few studies have been performed to examine antagonistic or synergistic effects when two or more treatments are combined, and more scientific research is also necessary for assessing the efficacy of preventive measures, and the potential of an attract-and-kill strategy. Further research on the effect of treatments under field conditions is also necessary as very different results are often obtained in the field compared to a laboratory environment.

In addition, IPM focusses on non-chemical treatments, but EU legislation highly limits the development and the registration of green products and feed additives. With the growing concern of the society related to the negative effects of synthetic pesticides, the EU legislation should search for opportunities for an increased introduction of registered green and safe products on the market.

More knowledge on resistance emergence against chemical and biological tactics and behavioral adaptations to certain treatments and conditions is also indispensable for further development of efficient and sustainable IPM strategies.

Finally, chemical pesticides can be part of an IPM strategy though *D. gallinae-*specific (chemical) pesticides are currently not available and should be developed.

Ideally, some clearly-defined schedules of IPM strategies would be developed that farmers simply need to follow and implement. An attempt was made by Dutch farmers, farm advisers and researchers, making a so-called “*Farm plan for control of PRM at layers*” based on all IPM steps (41). As these farmers found out, and as explained above, a single optimal strategy does not exist as various parameters within poultry houses can affect the efficacy of treatments and the population dynamics of *D. gallinae*. Mul et al. (72) set the basis for a house-specific treatment advice algorithm, indicating the most cost effective point in time for a treatment. This algorithm is based on a house-specific mite population forecasting model (72) and a house-specific economic model. The models and algorithm were tested at three commercial layer houses and were here able to indicate the point in time for a cost effective treatment to return the mite population to a certain level in the layer house. Further fine-tuning and testing of these models would provide useful tools for IPM implementation on farms. The reasons why some farms only have minor problems with *D. gallinae*, while others suffer from large infestations, even despite several control actions are often unclear. Identifying the reason(s) why some layer houses have lower population growths would provide valuable information.

The aim should thus be to develop dynamic IPM strategies, with different options under different circumstances (see “Evaluation”). Also in horticulture, IPM strategies are composed in such a way, with farmers often hiring IPM advisors for a continuous follow-up and counsel regarding the IPM measures and strategy. The current review highlights which options are available within each IPM step for the control of *D. gallinae* in layer houses, and which important knowledge gaps still need to be tackled to develop practical and efficient IPM strategies, with guidance of advisors. Although this review focusses on layer houses, similar approaches can and should be used in breeder and rearing farms where *D. gallinae* infestations also occur and from which infestations can be brought into a layer house. Indeed, for efficient control, the whole egg production chain needs to be taken into account (19). Further, the current review focusses on the control of *D. gallinae* in European layer houses, but the information can similarly be used for farms outside the EU. Ultimately, control of other pest species in layer houses could be integrated in the strategies.

## Author Contributions

ED wrote the main text, with input from all co-authors. NS did the final proofreading.

## Conflict of Interest

JW was employed by the company RSK ADAS, Ltd. AV is employed by the company Koppert BV. The remaining authors declare that the research was conducted in the absence of any commercial or financial relationships that could be construed as a potential conflict of interest.

## References

[1] Chauve CM. The poultry red mite *Dermanyssus gallinae* (De Geer, 1778) : current situation and future prospects for control. Vet Parasitol. (1998) 79:239–45. 10.1016/S0304-4017(98)00167-8 9823064

[2] Moss WW. An illustrated key to the species of the acarine genus *Dermanyssus* (Mesostigmata: Laelapoidea: Dermanyssidae). J Med Entomol. (1968) 5:67–84. 10.1093/jmedent/5.1.67 5644463

[3] Di Palma A Giangaspero A Cafiero MA Germinara GS. A gallery of the key characters to ease identification of *Dermanyssus gallinae* (acari: gamasida: dermanyssidae) and allow differentiation from *ornithonyssus sylviarum* (acari: gamasida: macronyssidae). Parasit Vect. (2012) 5:1–10. 10.1186/1756-3305-5-104 22647594 PMC3419681

[4] Sikes RK Chamberlain RW. Laboratory observations on three species of bird mites. Parasitology. (1954) 40:691–7. 10.2307/3273713 13212549

[5] Axtell RC Arends JJ. Ecology and management of arthropod pests of poultry. Annu Rev Entomol. (1990) 35:101–26. 10.1146/annurev.en.35.010190.000533 2405769

[6] Maurer V Bieri M Folsch DW Das suchverhalten von *Dermanyssus gallinae* in hühnerställen. Host-finding of Derman.yssus gallinae in poultry houses. Arch Geflügelkd. (1988) 52:209–15.

[7] George DR Finn RD Graham KM Mul MF Maurer V Moro CV. Should the poultry red mite *Dermanyssus gallinae* be of wider concern for veterinary and medical science? Parasit Vect. (2015) 8:178. 10.1186/s13071-015-0768-7 25884317 PMC4377040

[8] Sparagano OAE George DR Harrington DWJ Giangaspero A. Significance and control of the poultry red mite, *Dermanyssus gallinae*. Annu Rev Entomol. (2014) 59:447–66. 10.1146/annurev-ento-011613-162101 24397522

[9] Tomley FM Sparagano O. Spotlight on avian pathology: red mite, a serious emergent problem in layer hens. Avian Pathol. (2018) 47:1–9. 10.1080/03079457.2018.1490493 29954185

[10] Kilpinen O Roepstorff A Permin A Nørgaard-Nielsen G Lawson LG Simonsen HB. Influence of *Dermanyssus gallinae* and Ascaridia galli infections on behaviour and health of laying hens (Gallus gallus domesticus). Br Poult Sci. (2005) 46:26–34. 10.1080/00071660400023839 15835249

[11] Valiente Moro C De Luna CJ Tod A Guy JH Sparagano OA Zenner L. The poultry red mite (*Dermanyssus gallinae*) : a potential vector of pathogenic agents. In: O. Sparagano, editor. Control of Poultry Mites (Dermanyssus). Dordrecht: Springer (2009). p. 93–104. 10.1007/978-90-481-2731-3_10 19205905

[12] Sparagano OAE Giangaspero A Parasitism in egg production systems: the role of the red mite (*Dermanyssus gallinae*). In: Nys Y, Bain M, Van Immerseel F, editors. Improving the Safety Quality of Eggs Egg Products. Cambridge: Woodhead Publishing (2011). p. 394–414. 10.1533/9780857093912.3.394

[13] Pavličević A Pavlović I Ratajac R Popović D Davidović B Krnjajić D Poultry welfare in terms of poultry red mite (*Dermanyssus gallinae*) impact and control. Biotechnol Anim Husbandry. (2019) 35:1–11. 10.2298/BAH1901001P

[14] Cafiero MA Barlaam A Camarda A Radeski M Mul M Sparagano O. *Dermanysuss gallinae* attacks humans. Mind the gap! Avian Pathol. (2019) 48 (Suppl. 1): S22–S34. 10.1080/03079457.2019.1633010 31264450

[15] Van Emous R Verwachte Schade Bloedluis 21 Miljoen Euro. (2017). Available online at: https://www.pluimveeweb.nl/artikel/163578-verwachtte-schade-bloedluis-21-miljoen-euro/ (accessed April 24, 2020).

[16] Sleeckx N Van Gorp S Koopman R Kempen I Van Hoye K De Baere K. Production losses in laying hens during infestation with the poultry red mite *Dermanyssus gallinae*. Avian Pathol. (2019) 48 (Suppl. 1): S17–S21. 10.1080/03079457.2019.1641179 31298932

[17] Van Emous R RA van Niekerk TGCM Mul M E 11 miljoen schade voor de sector : enquête vogelmijten op leghennenbedrijven. De Pluimveehouderij. (2005) 358–9.

[18] Marangi M Morelli V Pati S Camarda A Cafiero MA Giangaspero A. Acaricide residues in laying hens naturally infested by red mite *Dermanyssus gallinae*. PLoS ONE. (2012) 7:e31795. 10.1371/journal.pone.0031795 22363736 PMC3283649

[19] Mul M van Niekerk T Chirico J Maurer V Kilpinen O Sparagano O Control methods for *Dermanyssus gallinae* in systems for laying hens: results of an international seminar. World Poultry Sci J. (2009) 65:589–600. 10.1017/S0043933909000403

[20] Arends JJ Robertson SH Integrated pest management for poultry production: implementation through integrated poultry companies. Poultry Sci. (1986) 65:675–82. 10.3382/ps.0650675

[21] Harrington DWJ George DR Guy JH Sparagano OAE Opportunities for integrated pest management to control the poultry red mite, *Dermanyssus gallinae*. World Poultry Sci J. (2011) 67:83–94. 10.1017/S0043933911000079

[22] Sparagano O George DR Finn RD Giangaspero A Mul MF Papadopoulos E *Dermanyssus gallinae* and poultry production: Impact, management, and a predicted compatibility matrix for integrated approaches. In: Conference Information and Proceedings of the XIVth European Poultry Conference Stavanger (2014). p. 131–41.

[23] Mul MF van Riel JW Meerburg BG Dicke M George DR Groot Koerkamp PWG. Validation of an automated mite counter for *Dermanyssus gallinae* in experimental laying hen cages. Exp Appl Acarol. (2015) 66:589–603. 10.1007/s10493-015-9923-2 26002308 PMC4481303

[24] Barzman M Bàrberi P Birch ANE Boonekamp P Dachbrodt-Saaydeh S Graf B Eight principles of integrated pest management. Agronomy Sustain Dev. (2015) 35:1199–215. 10.1007/s13593-015-0327-9

[25] Peshin R Bandra RS Zhang W Wilson L Dhawan AK Integrated pest management: A global overview of history, programs and adoption. In: Peshin RD, Asho L, editors. Integrated Pest Management Volume 1: Innovation-Development Process. Dordrecht: Springer (2009). p. 1–49. 10.1007/978-1-4020-8992-3_1

[26] ANSES French Agancy for Food, Environmental and Occupational Health and Safety. (2000). Available online at: https://www.anses.fr/en/content/anses-and-biological-pest-control. (accessed August 07, 2020).

[27] Fasanmi F Onyima VC Current concepts in the control of ticks and tick-borne diseases in Nigeria—A review. Int J Trop Insect Sci. (1992) 13:615–9. 10.1017/S1742758400016210

[28] Van Wyk JA Hoste H Kaplan RM Besier RB. Targeted selective treatment for worm management—how do we sell rational programs to farmers? Vet Parasitol. (2006) 139:336–46. 10.1016/j.vetpar.2006.04.023 16774807

[29] Axtell RC Livestock integrated pest management (IPM): principles prospects. In. Knapp FW, editor. Systems Approach to Animal Health and Production Lexington: University of Kentucky (1981). p. 31–40.

[30] Maurer V Perler E Heckendorn F. *In vitro* effects of oils, silicas and plant preparations against the poultry red mite *Dermanyssus gallinae*. Exp Appl Acarol. (2009) 48:31–41. 10.1007/978-90-481-2731-3_5 19229641

[31] Roy L Chauve CM Buronfosse T Contrasted ecological repartition of the northern fowl mite *ornithonyssus sylviarum* (mesostigmata: Macronyssidae) and the chicken red mite *Dermanyssus gallinae* (mesostigmata: Dermanyssidae). Acarologia. (2010) 50:207–19. 10.1051/acarologia/20101958

[32] Ciloglu A Yildirim A Onder Z Yetismis G Duzlu O Simsek E Molecular characterization of poultry red mite, *Dermanyssus gallinae* lineages in Turkey and first report of plasmodium species in the mite populations. Int J Acarol. (2020) 46:241–6. 10.1080/01647954.2020.1758775

[33] Maurer V Baumgärtner J Bieri M Fölsch DW The occurrence of the chicken mite *Dermanyssus gallinae* (acari: dermanyssidae) in swiss poultry houses. Mitt Schweiz Entomol Ges. (1993) 66:87–97.

[34] Ribbens S Dewulf J Koenen F Mintiens K De Sadeleer L de Kruif A. A survey on biosecurity and management practices in Belgian pig herds. Prev Vet Med. (2008) 83:228–41. 10.1016/j.prevetmed.2007.07.009 17850906

[35] Sylejmani D Musliu A Ramadani N Sparagano O Hamidi A. *Associations* between the level of biosecurity and occurrence of *Dermanyssus gallinae* and *Salmonella* spp. in layer farms *Avian Dis*. (2016) 60:454–9. 10.1637/11327-111415-Reg 27309287

[36] Mul MF Koenraadt CJ. Preventing introduction spread of *Dermanyssus gallinae* in poultry facilities using the HACCP method. In: Sparagano O, editor. Control of Poultry Mites (Dermanyssus). Dordrecht: Springer (2009). p. 167–81. 10.1007/978-90-481-2731-3_15 19221882

[37] Øines Ø Brännström S Molecular investigations of cytochrome c oxidase subunit I (COI) and the internal transcribed spacer (ITS) in the poultry red mite, *Dermanyssus gallinae*, in northern Europe and implications for its transmission between laying poultry farms. Med Vet Entomol. (2011) 25:402–12. 10.1111/j.1365-2915.2011.00958.x 21501200

[38] Roy L Buronfosse T. Using mitochondrial and nuclear sequence data for disentangling population structure in complex pest species: a case study with *Dermanyssus gallinae*. PLoS.ONE. (2011) 6:e22305. 10.1371/journal.pone.0022305 21799818 PMC3143145

[39] Wytynck W Venken J Laat de bloedluis je bedrijfsresultaat niet leegzuigen! Bloedluizen herkennen, voorkomen en bestrijden. Geel: Boerenbond & Provinciale vereniging voor bedrijfspluimveehouders (2013).

[40] MSD Animal health. (2000). Available online at: https://www.exzolt.com/welfare/biosecurity.aspx. (accessed August 06, 2020).

[41] Mul MF Neijenhuis F van Niekerk TGCM Aanpak van vogelmijt bij pluimvee. Project report 1226. Wageningen: Wageningen University & Research (2020). 10.18174/512820

[42] Tucci EC Prado AP Araujo RP. Development of *Dermanyssus gallinae* (Acari: Dermanyssidae) at different temperatures. Vet. Parasitol. (2008) 155:127–32. 10.1016/j.vetpar.2008.04.005 18502586

[43] Van Emous RV Fiks TGCM Mul MF Bloedluizen (vogelmijten) op papier en in de praktijk. Anim Sci Group. (2005) 17:42.

[44] Kilpinen O. Activation of the poultry red mite, *Dermanyssus gallinae* (Acari: Dermanyssidae), by increasing temperatures. Exp Appl Acarol. (2001) 25:859–67. 10.1023/A:1020409221348 12455876

[45] Zoons J Deschuytere H DERMANYSSUS: potentiële bestrijdingsmethoden van Dermanyssus gallinae bij pluimvee in de praktijk. Brussels: Project of the Federal Agency for the Safety of the Food Chain (2018).

[46] Hoffman GV. Vogelmilben als lastlinge, krankenheitsserzeuger und vektoren bei mensch und nutztier. Dtsch Tieraerztl Wochenschr. (1987) 95:7–10. 3281818

[47] Huber K Zenner L Bicout DJ. Modelling population dynamics and response to management options in the poultry red mite *Dermanyssus gallinae* (Acari: Dermanyssidae). Vet Parasitol. (2011) 176:65–73. 10.1016/j.vetpar.2010.10.043 21093987

[48] Chiron G Varescon A Lubac S Bicout DJ Roy L A decision-making method to anticipate outbreaks of *Dermanyssus gallinae* populations in layer farms. Actes des 11èmes Journées de la Recherche Avicole et Palmipèdes à Foie Gras, Tours, France, les 25 et 26 mars. (2015) 2015:179–83.

[49] Mul MF Ploegaert JP George DR Meerburg BG Dicke M Koerkamp PWG Structured design of an automated monitoring tool for pest species. Biosyst Eng. (2016) 151:126–40. 10.1016/j.biosystemseng.2016.08.023

[50] Lammers GA Bronneberg RGG Vernooij JCM Stegeman JA. Experimental validation of the AviVet trap, a tool to quantitatively monitor the dynamics of *Dermanyssus gallinae* populations in laying hens. Poult Sci. (2017) 96:1563–72. 10.3382/ps/pew428 27920194

[51] Kirkwood A. Longevity of the mites Dermanyssus gallinae and Liponyssus sylviarum. Exp Parasitol. (1963) 14:358–66. 10.1016/0014-4894(63)90043-2 14099848

[52] Tucci EC Bruno TV Guimarães JH Armadilha para amostragem de Dermanyssus gallinae (Acari Dermanyssidae) em aviários de postura comercial. Arquivos Instit Biol. (1988) 56:114

[53] Nordenfors H Höglund J Uggla A. Effects of temperature and humidity on oviposition, molting, and longevity of *Dermanyssus gallinae* (Acari: Dermanyssidae). J Med Entomol. (1999) 36:68–72. 10.1093/jmedent/36.1.68 10071495

[54] Van Emous RA ten Napel J Monitoring van bloedluispopulatie op praktijkbedrijven; buis met stokje zeer geschikt voor bewustwording. De Pluimveehouderij. (2007) 37:8–9.

[55] Pavliĉević A Pavlović I Stajković N Method for early detection of poultry red mite Dermanyssus gallinae (De Geer, 1778). Biotechnol Anim Husband. (2007) 23:119–27. 10.2298/BAH0704119P

[56] Zenner L Bon G Chauve C Nemoz C Lubac S Monitoring of *Dermanyssus gallinae* in free-range poultry farms. In: Sparagano O, editor. Control of Poultry Mites (Dermanyssus). Dordrecht: Springer (2009). p. 157–166. 10.1007/978-90-481-2731-3_14 19252824

[57] Cox M De Baere K Vervaet E Zoons J Fiks-Van Niekerk T Red mites: monitoring method and treatment. In: Book of Abstracts 8th European Symposium on Poultry Welfare. Cervia (2009). p. 18–22.

[58] Tuovinen T Heikkilä P Juvonen S Lindqvist B Tuovinen T Kanapunkki Hallintaan Munintakanaloissa.Control of Red Poultry Mite in Laying Hen Houses. Research report MMM12-3/312/2010. Joikioinen: MTT Kasvintuotannon tutkimus (2010).

[59] Mozafar F Tackling red mite in laying hens remains a challenge. Poultry World. (2014) 30:22–24

[60] Schulz J Massnahmen zur Bekämpfung der Roten Voleglmilbe (Dermanyssus gallinae) in der ökologischen Legehennenhaltung. (Ph.D. thesis). Freie Universität Berlin, Berlin, Germany (2014).

[61] Roy L Chiron G Lubac S Bicout DJ Tape-traps as an easy-to-use tool for monitoring and surveillance of the poultry red mite in cage and free-range layer farms. In: XIVth European Poultry Conference, Stavanger (Norway), June 23–27th 2014. (2014).

[62] Dick van de Ven Q-Perch, electronic control of red mite. Vencomatic Group. In: Abstarct Book, 2nd COST ACTION FA1404. Improving Current Understanding and Research for Sustainable Control of the Poultry Red Mite Dermanyssus gallinae (COREMI), 1st−3rd June, Zagreb, Croatia. (2016). p. 24

[63] Sokol R Koziatek Sadlowska S Monitoring the invasion of Dermanyssus gallinae in flocks of layer hens. In: Final programme and Book of Abstract of the 2nd COST Conference and Management Committee (MC) Meeting, COST Action FA1404 Improving Current Understanding and Research for Sustainable Control of the Poultry Red Mite Dermanyssus gallinae (COREMI), Zagreb (2016). p. 24

[64] Dovc A Semrov N Vergles Rataj A Lindtner Knific R Nemec M Trbovsek T Evaluation of alternative method for sampling Dermanyssus gallinae (Acari: Dermanyssidae) in poultry farms. In: Final Programme and Book of Abstract of the 2nd COST Conference and Management Committee (MC) Meeting, COST Action FA1404 Improving Current Understanding and Research for Sustainable Control of the Poultry Red Mite Dermanyssus gallinae (COREMI), Zagreb (2016). p. 23.

[65] Nordenfors H Chirico J. Evaluation of a sampling trap for *Dermanyssus gallinae* (Acari: Dermanyssidae). J Econ Entomol. (2001) 94:1617–21. 10.1603/0022-0493-94.6.1617 11777073

[66] Schneider CA Rasband WS Eliceiri KW. NIH Image to Image J: 25 years of image analysis, *Nat Methods*. (2012) 9:671–75. 10.1038/nmeth.2089 22930834 PMC5554542

[67] Chirico J Tauson R. Traps containing acaricides for the control of *Dermanyssus gallinae*. Vet Parasitol. (2002) 110:109–16. 10.1016/S0304-4017(02)00310-2 12446095

[68] Nascimento MM Alves LFA de Oliveira DGP Lopes RB Guimarães ATB. Laboratory and field evaluation of an autoinoculation device as a tool to manage poultry red mite, *Dermanyssus gallinae*, infestations with Beauveria bassiana. Exp Appl Acarol. (2020) 80:151–65. 10.1007/s10493-020-00466-6 31950300

[69] Sleeckx N Kempen I Zoons J The potential of an integrated PRM strategy in practice. In: Sparagano et al., editors. Improving Current Understanding and Research for Sustainable Control of the Poultry Red Mite Dermanyssus gallinae (COREMI). 3rd COST Conference COREMI., 19-22 September, Oeiras, Portugal. (2017). p. 31.

[70] Koenraadt CJM Dicke M. The role of volatiles in aggregation and host-seeking of the haematophagous poultry red mite *Dermanyssus gallinae* (Acari: Dermanyssidae). Exp Appl Acarol. (2010) 50:191–9. 10.1007/s10493-009-9305-8 19760508

[71] COREMI Cost Action Network (FA1404). Improving current understandingand research for sustainable control of the poultry red mite *Dermanyssus gallinae* Available online at: www.coremi.eu. (2000).

[72] Mul MF van Riel JW Roy L Zoons J André G George DR. Development of a model forecasting *Dermanyssus gallinae*'s population dynamics for advancing integrated pest management in laying hen facilities. Vet Parasitol. (2017) 245:128–40. 10.1016/j.vetpar.2017.07.027 28969831

[73] Mul MF. Advancing integrated pest management for Dermanyssus gallinae in laying hen facilities. (Ph.D. Thesis). Wageningen University, Wageningen, Netherlands. (2017). p. 194.

[74] George DR Guy JH Arkle S Harrington D De Luna C Okello EJ Use of plant-derived products to control arthropods of veterinary importance: a review. Ann N Y Acad Sci. (2008) 1149:23–6. 10.1196/annals.1428.021 19120167

[75] George DR Sparagano OAE Port G Okello E Shiel RS Guy JH. Repellence of plant essential oils to *Dermanyssus gallinae* and toxicity to the non-target invertebrate *Tenebrio molitor*. Vet Parasitol. (2009) 162:129–34. 10.1016/j.vetpar.2009.02.009 19264408

[76] George DR Sparagano OAE Port G Okello E Shiel RS Guy JH The effect of essential oils showing acaricidal activity against the poultry red mite (*Dermanyssus gallinae*) on aspects of welfare and production of laying hens. Animal Welfare. (2010) 19:265–73. 10.1016/j.vetpar.2009.12.038

[77] Kim SI Na YE Yi JH Kim BS Ahn YJ. Contact and fumigant toxicity of oriental medicinal plant extracts against *Dermanyssus gallinae* (Acari: Dermanyssidae). Vet Parasitol. (2007) 145:377–82. 10.1016/j.vetpar.2006.12.021 17289270

[78] Bartley K Turnbull F Wright HW Huntley JF Palarea-Albaladejo J Nath M. Field evaluation of poultry red mite (*Dermanyssus gallinae*) native and recombinant prototype vaccines. Vet Parasitol. (2017) 244:25–34. 10.1016/j.vetpar.2017.06.020 28917313 PMC5613835

[79] Lesna I Wolfs P Faraji F Roy L Komdeur J Sabelis MW. Candidate predators for biological control of the poultry red mite *Dermanyssus gallinae*. In: Sparagano O, editors. Control of Poultry Mites (Dermanyssus), Dordrecht: Springer (2009). p. 63–80. 10.1007/978-90-481-2731-3_8 19184469

[80] Roy L El Adouzi M Moraza ML Chiron G de Janti EV Le Peutrec G (2017) Arthropod communities of laying hen houses: an integrative pilot study toward conservation biocontrol of the poultry red mite *Dermanyssus gallinae*. Biol Control. 114:176–94.s. 10.1016/j.biocontrol.2017.08.006

[81] Oliveira DGP Alves LFA Sosa-Gomez DR Advancesand perspectives of the use of the entomopathogenic fungi *Beauveria bassiana* and *Metarhizium anisopliae* for the control of arthropod pests in poultry production. Br J Poultry Sci. (2014) 16:1–12. 10.1590/S1516-635X2014000100001

[82] Axtell RC Poultry integrated pest management; status and future. Integr Pest Manag Rev. (1999) 4:53–73. 10.1023/A:1009637116897

[83] De Luna CJ Moro CV Guy JH Zenner L Sparagano OA. Endosymbiotic bacteria living inside the poultry red mite (*Dermanyssus gallinae*). Exp Appl Acarol. (2009) 48:105–13. 10.1007/978-90-481-2731-3_11 19145467

[84] Kilpinen O Steenberg T. Inert dusts and their effects on the poultry red mite (*Dermanyssus gallinae*). Exp Appl Acarol. (2009) 48:51–62. 10.1007/978-90-481-2731-3_7 19160061

[85] Schulz J Berk J Suhl J Schrader L Kaufhold S Mewis I. Characterization, mode of action, and efficacy of twelve silica-based acaricides against poultry red mite (*Dermanyssus gallinae*) *in vitro*. Parasitol Res. (2014) 113:3167–75. 10.1007/s00436-014-3978-6 24908434

[86] Birkett MA Hassanali A Hoglund S Pettersson J Pickett JA. Repellent activity of catmint, nepeta cataria, and iridoid nepetalactone isomers against afro-tropical mosquitoes, ixodid ticks and red poultry mites. Phytochemistry. (2011) 72:109–14. 10.1016/j.phytochem.2010.09.016 21056438

[87] Gay M Lempereur L Francis F Megido RC. Control of Dermanyssus gallinae (De Geer 1778) and other mites with volatile organic compounds, a review. Parasitology. (2020) 147:731–9. 10.1017/S0031182020000530 32312341 PMC10318255

[88] Ellse L Wall R. The use of essential oils in veterinary ectoparasite control: a review. Med Vet Entomol. (2014) 28:233–43. 10.1111/mve.12033 24147451

[89] Kim SI Yi JH Tak JH Ahn YJ. Acaricidal activity of plant essential oils against Dermanyssus gallinae (Acari: Dermanyssidae). Vet Parasitol. (2004) 120:297–304. 10.1016/j.vetpar.2003.12.016 15063940

[90] Na YE Kim SI Bang HS Kim BS Ahn YJ. Fumigant toxicity of cassia and cinnamon oils and cinnamaldehyde and structurally related compounds to *Dermanyssus gallinae* (Acari: Dermanyssidae). Vet Parasitol. (2011) 78:324–9. 10.1016/j.vetpar.2011.01.034 21324598

[91] Lee SJ Kim HK Kim GH Toxicity and effects of essential oils and their components on *Dermanyssus gallinae* (Acari: Dermanyssidae). Exp Appl Acarol. (2019) 78:65–78. 10.1007/s10493-019-00363-7 31069572

[92] López MD Pascual-Villalobos MJ Mode of inhibition of acetylcholinesterase by monoterpenoids and implications for pest control. Indus Crops Prod. (2010) 31:284–8. 10.1016/j.indcrop.2009.11.005

[93] Sparagano O Khallaayoune K Duvallet G Nayak S George D. comparing terpenes from plant essential oils as pesticides for the poultry red mite (*Dermanyssus gallinae*). Transboundary Emerg Dis. (2013) 60:150–3. 10.1111/tbed.12138 24589115

[94] George DR Sparagano OAE Port G Okello E Shiel RS Guy JH. Environmental interactions with the toxicity of plant essential oils to the poultry red mite *Dermanyssus gallinae*. Med Vet Entomol. (2010) 24:1–8. 10.1111/j.1365-2915.2009.00855.x 20377725

[95] Abdel-Ghaffar F Sobhy HM Al-Quraishy S Semmler M. Field study on the efficacy of an extract of neem seed (Mite-Stop®) against the red mite *Dermanyssus gallinae* naturally infecting poultry in Egypt. Parasitol Res. (2008) 103:481–5. 10.1007/s00436-008-0965-9 18481087

[96] Abdel-Ghaffar F Semmler M Al-Rasheid K Mehlhorn H. *In vitro* efficacy of ByeMite® and Mite-Stop® on developmental stages of the red chicken mite *Dermanyssus gallinae*. Parasitol Res. (2009) 105:1469. 10.1007/s00436-009-1590-y 19680689

[97] Locher N Al-Rasheid KA Abdel-Ghaffar F Mehlhorn H. *In vitro* and field studies on the contact and fumigant toxicity of a neem-product (Mite-Stop®) against the developmental stages of the poultry red mite *Dermanyssus gallinae*. Parasitol Res. (2010) 107:417–23. 10.1007/s00436-010-1882-2 20424858

[98] Wright HW Bartley K Nisbet AJ McDevitt RM Sparks NH Brocklehurst S. The testing of antibodies raised against poultry red mite antigens in an *in vitro* feeding assay; preliminary screen for vaccine candidates. Exp Appl Acarol. (2009) 48:81. 10.1007/s10493-009-9243-5 19184466

[99] Bartley K Wright HW Huntley JF Manson ED Inglis NF McLean K. Identification and evaluation of vaccine candidate antigens from the poultry red mite (*Dermanyssus gallinae*). Int J Parasitol. (2015) 45:819–30. 10.1016/j.ijpara.2015.07.004 26296690 PMC4655837

[100] Pritchard J Kuster T Sparagano O Tomley F. Understanding the biology and control of the poultry red mite *Dermanyssus gallinae*: a review. Avian Pathol. (2015) 44:143–53. 10.1080/03079457.2015.1030589 25895578

[101] Schicht S Qi W Poveda L Strube C Whole transcriptome analysis of the poultry red mite *Dermanyssus gallinae* (De Geer, 1778). Parasitology. (2014) 141:336–46. 10.1017/S0031182013001467 24135293

[102] Burgess STG Bartley K Nunn F Wright HW Hughes M Gemmell M. Draft genome assembly of the poultry red mite, *Dermanyssus gallinae*. Microbiol Resour Announc. (2018) 8:e01221–18. 10.1128/MRA.01221-18 30533782 PMC6256547

[103] Harrington D Canales M de la Fuente J de Luna C Robinson K Guy J. Immunisation with recombinant proteins subolesin and Bm86 for the control of *Dermanyssus gallinae* in poultry. Vaccine. (2009) 27:4056–63. 10.1016/j.vaccine.2009.04.014 19501789

[104] Zriki G Blatrix R Roy L. Predation interactions among henhouse-dwelling arthropods, with a focus on the poultry red mite *Dermanyssus gallinae*. Pest Manag Sci. (2020) 76:3711–9. 10.1002/ps.5920 32431063

[105] Pilkington LJ Messelink G van Lenteren JC Le Mottee K “Protected *biological control*”–biological pest management in the greenhouse industry. Biol Control. (2010) 52:216–20. 10.1016/j.biocontrol.2009.05.022

[106] Tirry L Van Leeuwen T DERGAL: ‘Geïntegreerde plaagbestrijding van Dermanyssus gallinae bij leghennen. Project of the Federal Agency for the Safety of the Food Chain (Belgium). (2014)

[107] Steenberg T Kilpinen O Fungus infection of the chicken mite dermanyssus gallinae. IOBC WPRS Bull. Brussels (2003) 26:23−6.

[108] Kaoud HA Susceptibility of poultry red mites to entomopathogens. Int J Poult Sci. (2010) 9:259–63. 10.3923/ijps.2010.259.263

[109] Tavassoli M Allymehr M Pourseyed SH Ownag A Bernousi I Mardani K. Field bioassay of metarhizium anisopliae strains to control the poultry red mite *Dermanyssus gallinae*. Vet Parasitol. (2011) 178:374–78. 10.1016/j.vetpar.2011.01.031 21320753

[110] Immediato D Camarda A Iatta R Puttilli MR Ramos RAN Di Paola G. Laboratory evaluation of a native strain of Beauveria bassiana for controlling *Dermanyssus gallinae* (De Geer, 1778) (Acari: Dermanyssidae). Vet Parasitol. (2015) 212:478–82. 10.1016/j.vetpar.2015.07.004 26206607

[111] Tomer H Blum T Arye I Faigenboim A Gottlieb Y Ment D. Activity of native and commercial strains of Metarhizium spp. against the poultry red mite *Dermanyssus gallinae* under different environmental conditions. Vet Parasitol. (2018) 262:20–5. 10.1016/j.vetpar.2018.09.010 30389007

[112] Oliveira DGP Kasburg CR Alves LFA Efficacy of beauveria bassiana against the poultry red mite, *Dermanyssus gallinae* (De Geer, 1778) (Mesostigmata: Dermanyssidae), under laboratory and hen house conditions. Syst Appl Acarol. (2020) 25:895–905. 10.11158/saa.25.5.10

[113] Steenberg T Kilpinen O Moore D. Fungi for control of the poultry red mite, Dermanyssus gallinae. In: Proceeding International Workshop Implement Biocontrol Pract. Temp. Reg.—Present and Near Future. Flakkebjerg, Nov. 1–3, 2005. DIAS Rep. (2006) 119:71–74. 24253584

[114] Sokół R Szkamelski A Barski D. Influence of light and darkness on the behaviour of *Dermanyssus gallinae* on layer farms. Polish J Vet Sci. (2008) 11:71–73. 18540212

[115] Wang C Ma Y Huang Y Su S Wang L Sun Y. Darkness increases the population growth rate of the poultry red mite *Dermanyssus gallinae*. Parasites Vectors. (2019) 12:213. 10.1186/s13071-019-3456-1 31064400 PMC6505187

[116] Zoons J The effect of light programs on red mite (*Dermanyssus gallinae*) in battery cage housing. In: Perry GC, editor. Welfare of the Laying Hen. CABI Poultry Science Symposium Series Number 27. Wallingford: CAB (2004). p. 416.

[117] Stafford KA Lewis PD Coles GC. Preliminary study of intermittent lighting regimens for red mite (*Dermanyssus gallinae*) control in poultry houses. Vet Rec. (2006) 158:762–3. 10.1136/vr.158.22.762 16751311

[118] Merget R Bauer T Küpper H Philippou S Bauer H Breitstadt R. Health hazards due to the inhalation of amorphous silica. Arch Toxicol. (2002) 75:625–34. 10.1007/s002040100266 11876495

[119] Maurer V Perler E. (2006) Silicas for control of the poultry red mite *Dermanyssus gallinae*. In: Proceedings paper presented at Joint Organic Congress. Odense, Denmark.

[120] Alves LFA de Oliveira DGP Pares RB Sparagano OA Godinho RP. Association of mechanical cleaning and a liquid preparation of diatomaceous earth in the management of poultry red mite, *Dermanyssus gallinae* (Mesostigmata: Dermanyssidae). Exp Appl Acarol. (2020) 81:215–22 10.1007/s10493-020-00497-z 32378067

[121] Steenberg T Kilpinen O. Synergistic interaction between the fungus Beauveria bassiana and desiccant dusts applied against poultry red mites (Dermanyssus gallinae). Exp Appl Acarol. (2014) 62:511–24. 10.1007/s10493-013-9757-8 24253584

[122] Kilpinen O How to obtain a bloodmeal without being eaten by a host: the case of poultry red mite, *Dermanyssus gallinae*. Physiol Entomol. (2005) 30:232–40. 10.1111/j.1365-3032.2005.00452.x

[123] El Adouzi M Arriaga-Jiménez A Dormont L Barthes N Labalette A Lapeyre B. Modulation of feed composition is able to make hens less attractive to the poultry red mite Dermanyssus gallinae. Parasitology. (2019) 147:1–11. 10.1017/S0031182019001379 31559942 PMC10317670

[124] Roy L Arriaga-Jimenez A El Adouzi M Composition anti-acariens. Montpellier: Patent no WO2018109417A1; FR3060258A1 filed Dec 2016 issued May 2018 UPVM3, UCNRS M. SATT AxLR. (2018).

[125] Entrekin DL Oliver JH. Aggregation of the Chicken Mite, *Dermanyssus gallinae* (Acari: Dermanyssidae). J Med Entomol. (1982) 19:671–1. 10.1093/jmedent/19.6.671 7154024

[126] Birkett M Pickett J Dewhirst S Jespersen JB Steenberg T Kilpinen O Composition Comprising a Volatile Carbocyclic Acid or an Aldehyde and Its Use as Attractant for Mites. Slagelse: Patent no WO2010130990A8. Rothamsted Research Limited, Aarhus Universitet. (2010).

[127] Charmillot PJ Hofer D Pasquier D Attract and kill: a new method for control of the codling moth *Cydia pomonella*. Entomol Exp Appl. (2000) 94:211–6. 10.1046/j.1570-7458.2000.00621.x

[128] George DR Smith TJ Sparagano OAE Guy JH. The influence of ‘time since last blood meal'on the toxicity of essential oils to the poultry red mite (*Dermanyssus gallinae*). Vet Parasitol. (2008) 155:333–5. 10.1016/j.vetpar.2008.05.005 18565672

[129] Keïta A Pagot E Pommier P Baduel L Heine J Efficacy of phoxim 50% E.C. (ByeMite) for treatment of *Dermanyssus gallinae* in laying hens under field conditions. Re Med Vet Toulouse. (2006) 157:590–4.

[130] Meyer-Kuhling B Pfister K Müller-Lindloff J Heine J. Field efficacy of phoxim 50% (ByeMite) against the poultry red mite *Dermanyssus gallinae* in battery cages stocked with laying hens. Vet Parasitol. (2007) 147:289–96. 10.1016/j.vetpar.2007.04.012 17543456

[131] Gassel M Wolf C Noack S Williams H Ilg T The novel isoxazoline ectoparasiticide fluralaner: selective inhibition of rthropod gamma-aminobutyric acid- and Lglutamate- gated chloride channels and insecticidal/acaricidal activity. Insect Biochem Mol Biol. (2014) 45:111–24. 10.1016/j.ibmb.2013.11.009 24365472

[132] Brauneis MD Zoller H Williams H Zschiesche E Heckeroth AR. The acaricidal speed of kill of orally administered fluralaner against poultry red mites (*Dermanyssus gallinae*) on laying hens and its impact on mite reproduction. Parasites Vect. (2017) 10:594. 10.1186/s13071-017-2534-5 29197422 PMC5712167

[133] Thomas E Chiquet M Sander B Zschiesche E Flochlay AS. Field efficacy and safety of fluralaner solution for administration in drinking water for the treatment of poultry red mite (*Dermanyssus gallinae*) infestations in commercial flocks in Europe. Parasites Vect. (2017) 10:457. 10.1186/s13071-017-2390-3 28992814 PMC5632831

[134] Reich H Triacchini GA. Occurrence of residues of fipronil and other acaricides in chicken eggs and poultry muscle/fat. EFSA J. (2018) 16:e05164. 10.2903/j.efsa.2018.5164 32625889 PMC7009608

[135] Gokbulut C Ozuicli M Aslan B Aydin L Cirak VY. The residue levels of spinosad and abamectin in eggs and tissues of laying hens following spray application. Avian Pathol. (2019) 48 (Suppl. 1): S44–51. 10.1080/03079457.2019.1623380 31132863

[136] R4P Réseau de Réflexion et de Recherches sur lesRésistances aux Pesticides. (2020). Available online at: https://www.r4p-inra.fr/fr/home/ (accessed April 21, 2020).

[137] REX Consortium. The skill and style to model the evolution of resistance to pesticides and drugs. Evol Appl. (2010) 3:375–90. 10.1111/j.1752-4571.2010.00124.x 25567932 PMC3352466

[138] Rex Consortium. Heterogeneity of selection and the evolution of resistance. Trends Ecol Evol. (2013) 28:110–8. 10.1016/j.tree.2012.09.001 23040463

[139] Katsavou E Vlogiannitis S Karp-Tatham E Blake DP Ilias A Strube C. Identification and geographical distribution of pyrethroid resistance mutations in the poultry red mite *Dermanyssus gallinae*. Pest Manag Sci. (2020) 76:125–33. 10.1002/ps.5582 31400055

[140] Bartley K Wright HW Bull RS Huntley JF Nisbet AJ. Characterisation of *Dermanyssus gallinae* glutathione S-transferases and their potential as acaricide detoxification proteins. Parasites Vect. (2015) 8:350. 10.1186/s13071-015-0960-9 26112960 PMC4491418

[141] Roy L Chauve C Delaporte J Inizan G Buronfosse T. Exploration of the susceptibility of AChE from the poultry red mite *Dermanyssus gallinae* (Acari: Mesostigmata) to organophosphates in field isolates from France. In: Sparagano OAE. editor. Control of Poultry Mites (Dermanyssus). Dordrecht: Springer (2009). p. 19–30. 10.1007/978-90-481-2731-3_4 19214761

[142] Thind BB Ford HL. Assessment of susceptibility of the poultry red mite *Dermanyssus gallinae* (Acari: Dermanyssidae) to some acaricides using an adapted filter paper based bioassay. Vet Parasitol. (2007) 144:344–8. 10.1016/j.vetpar.2006.10.002 17157988

[143] Bardin M Ajouz S Comby M Lopez-Ferber M Graillot B Siegwart M. Is the efficacy of biological control against plant diseases likely to be more durable than that of chemical pesticides? Front Plant Sci. (2015) 6:566. 10.3389/fpls.2015.00566 26284088 PMC4515547

